# Supplementary material for: Drosophila motor neuron boutons remodel through membrane blebbing coupled with muscle contraction
Source: Nat Commun. 2023 Jun 8;14:3352. doi: 10.1038/s41467-023-38421-9 (PMC10250368; doi:10.1038/s41467-023-38421-9)
Supplement: Supplementary file 1 — Supplementary Information [file 41467_2023_38421_MOESM1_ESM.pdf]

Drosophila motor neuron boutons remodel through membrane blebbing coupled with muscle contraction

Andreia R. Fernandes[1], João P. Martins[1,2], Edgar R. Gomes[2], César S. Mendes[1] and Rita O. Teodoro[1]*

Supplementary Figures S1 – S25

Supplementary Figure 1

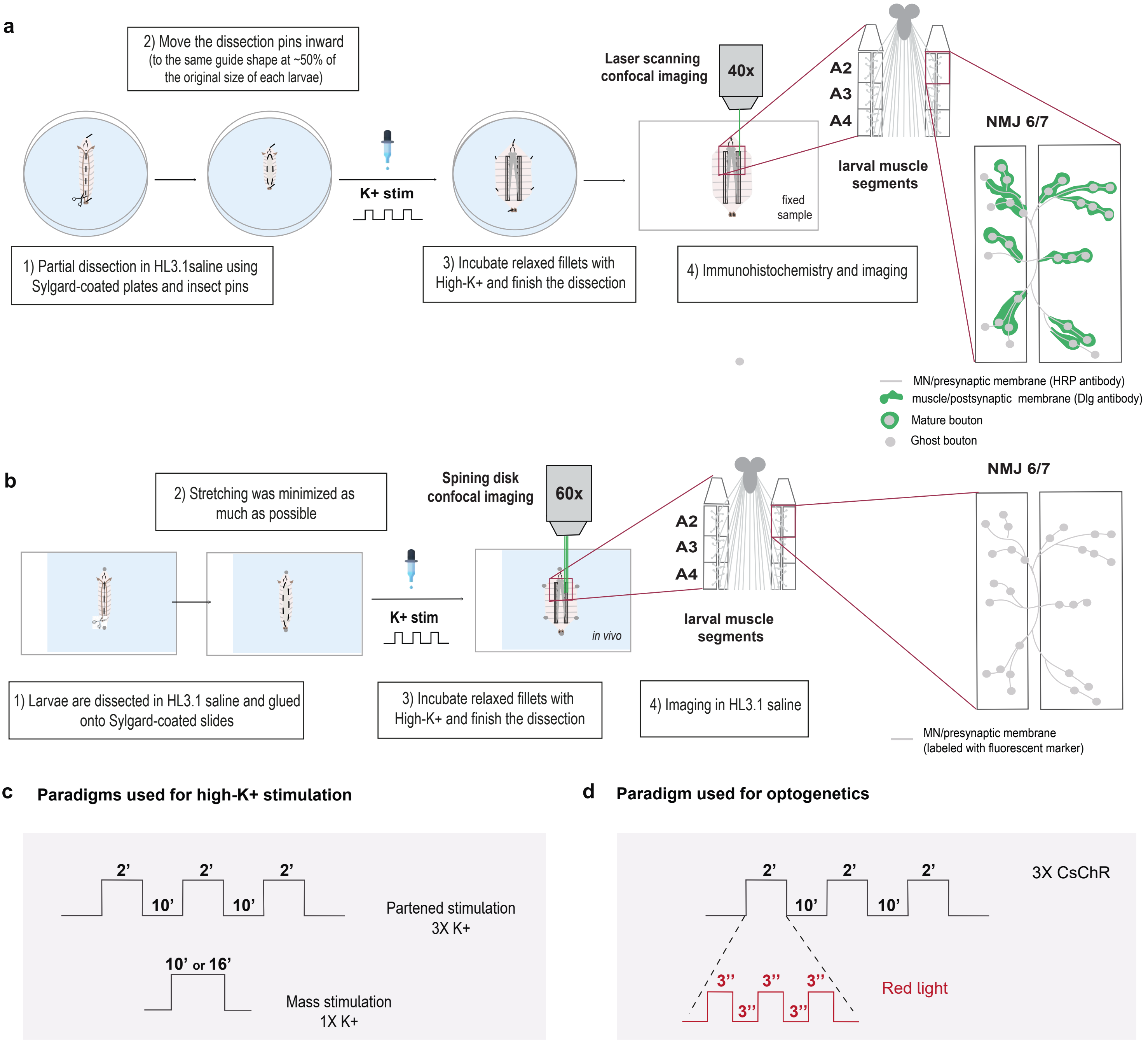

Supplementary Figure 1. Experimental methodology and stimulation paradigms used to elicit rapid activity-dependent plasticity at *Drosophila* NMJ. **a, b,** Schematics of the experimental setup, showing the methodology used throughout this manuscript. **a,** Laser scanning confocal imaging was used to quantify ghost bouton (GB) number in fixed samples. The presynaptic and postsynaptic membranes were labeled with antibodies against Horseradish peroxidase (HRP) and Disc-large (Dlg), respectively. GBs were identified by the lack of Dlg. **b,** Spinning disk confocal imaging is used to follow *in vivo* and in real-time bouton growth and remodeling of pre-existing boutons. Animals expressing fluorescent tags, CD4-Tomato (CD4-Tom, transmembranar) and monomeric Cherry (mCherry, cytosolic) under the control of NSyb-Gal4 and LexA Driver DVGlut, were used to visualize bouton growth dynamics. **c, d,** Activity-dependent plasticity stimulation protocols were performed on semi-dissected 3rd instar larvae, based on previous publications. **c,** Schematics of the stimulation protocols used, as described by Vasin *et al.* (2014) that uses a spaced high-K^+ depolarization paradigm (top) to induce patterned activity and fast bouton formation. All pulses consisted of a 2-min stimulation followed by 10-min rest periods for a total of 26-min per protocol, followed by 30-min rest prior to fixation, or directly to live imaging. Additionally, for live imaging, we also used a mass stimulation procedure of 10- or 16- min high-K^+ incubations developed by Martin *et al.* (2017). **d,** For the optogenetic stimulation protocol, we exposed transgenic animals expressing channelrhodopsin Chrimson (CsChrimson) channels in MNs to red light pulses. All pulses consisted of a 2-min stimulation (within each 2-min of stimulation, the larvae were exposed to rapid pulses of 3 s of lights on and 3 s of lights off) followed by 10-min rest periods for a total of 26-min per protocol, followed by 30-min rest prior to fixation, or live imaging.

Supplementary Figure 2

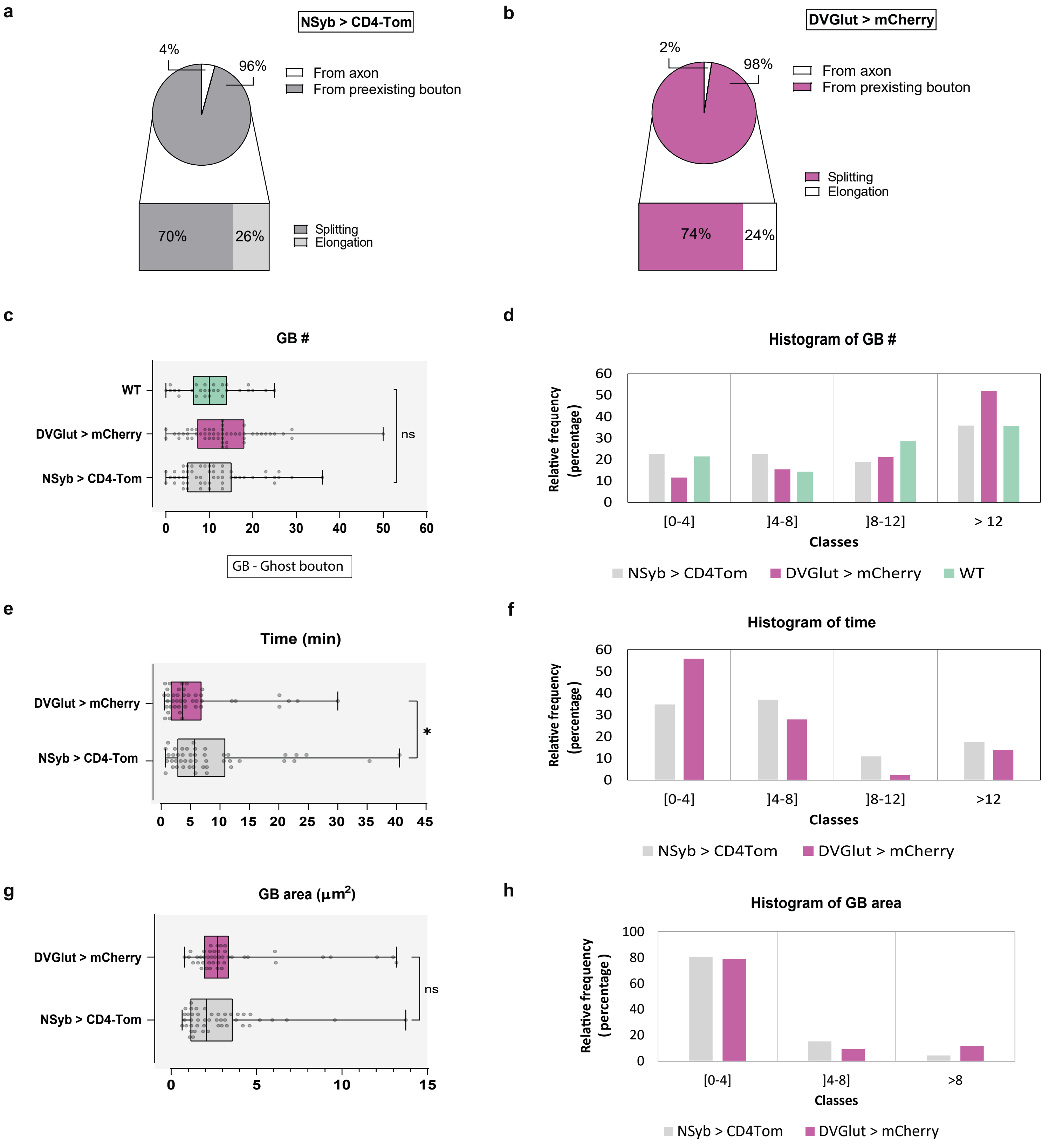

Supplementary Figure 2. Categorization of bouton formation based on origin and morphological alterations, and effect of membrane tags on bouton formation frequency and dynamics. **a, b,** Characterization of types of bouton formation based on the location from which they emerge (origin) and type of morphological alteration. **a,** Analysis of movies from 3rd instar larvae expressing CD4-Tomato (CD4-Tom, transmembranar) under the control of the neuronal driver NSyb-Gal4. 96% of boutons emerge from pre-existing boutons, 4% from the axon. From the boutons emerging from other boutons, 70% give rise to more boutons, and 26% go through a transition phase where two boutons are visible but that resolve into one, resulting in the increase in size of the mother bouton (elongation) (total of 46 boutons). **b**, analysis of movies from 3rd instar larvae expressing monomeric Cherry (mCherry, cytosolic) under the control of the driver DV-Glut-LexA. 98% of boutons emerge from pre-existing boutons, 2% from the axon. From the boutons emerging from other boutons, 74% give rise to more boutons, and 24% go through a transition phase where two boutons are visible but that resolve into one resulting in the increase in size of the mother bouton (elongation) (total of 43 boutons). Percentages are displayed considering the total number of boutons 100%. **c,** Boxplot (min to max) showing ghost bouton (GB) frequency between WT larvae (w^{1118}) and larvae expressing CD4-Tom or mCherry in neurons. Line is at median. All data points are plotted. **d,** Histogram showing percentage of GBs in each time class for the WT and membrane tags. N=28, 53 and 52 NMJs for WT, CD4-Tom and mCherry, respectively, from 3 biologically independent experiments. **e,** Boxplot (min to max) representing time of bouton formation; line is at median, this data showed that time of formation is slightly increased using CD4-Tom compared with mCherry (p=0.043). All data points are shown. **f,** Histogram showing percentage of bouton formation time in each class for both tags. **g,** Boxplot showing GB area when using CD4-Tom or mCherry. Line is at median. **h,** Histogram showing percentage of GBs in each area class for the tags used. All data points are presented. N=46 boutons (for CD4-Tom), and 43 boutons (for mCherry). All boxplots show 25th–75th percentiles, lines at the median, and whiskers from minimum to maximum. Statistical significance was determined using two-tailed non-parametric tests: Kruskal-Wallis test (in **c**) or Mann-Whitney test (in **e** and **g**); *p<0.05, ns is not significant. Source data provided as a Source Data file.

Supplementary Figure 3

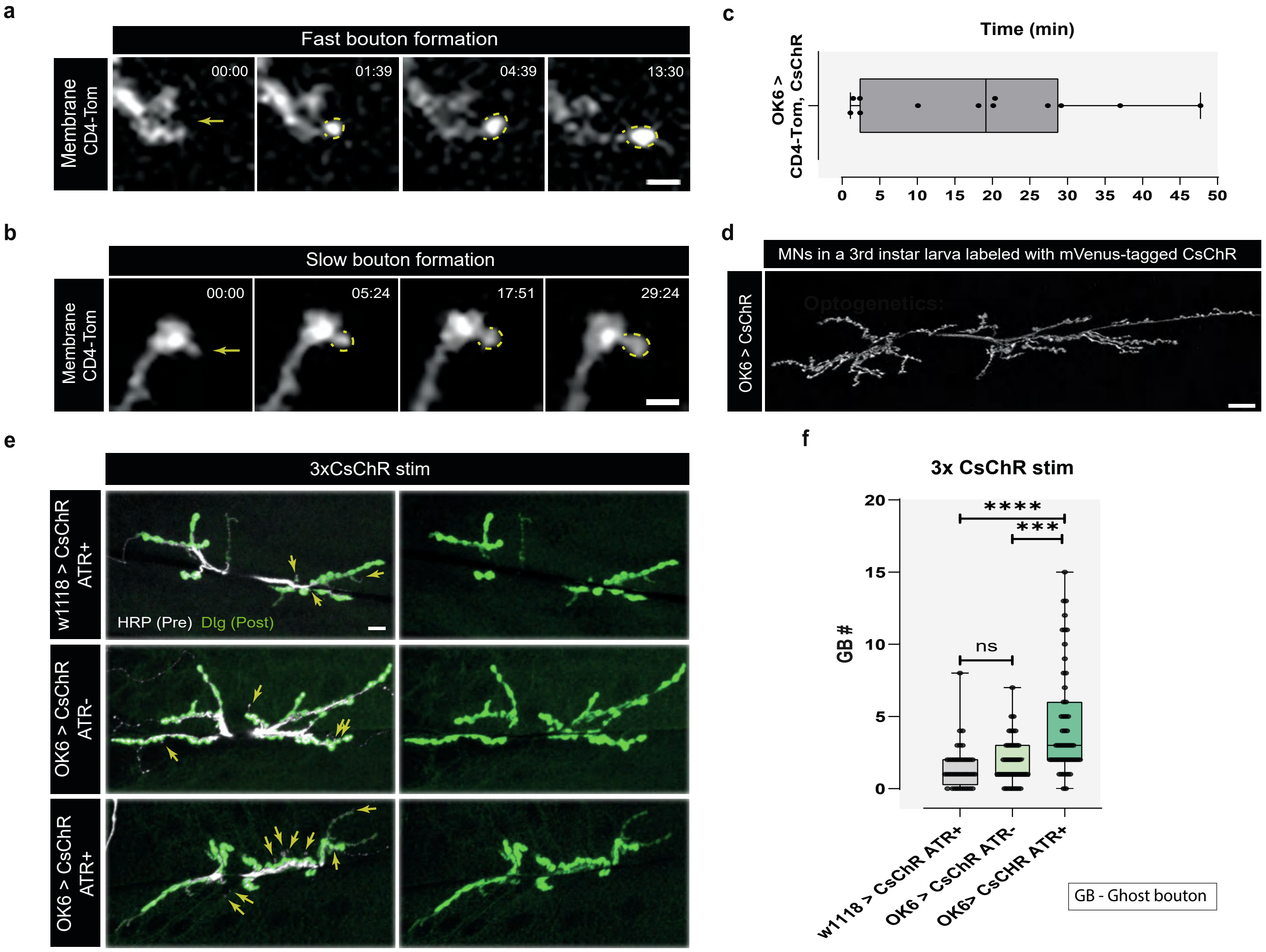

Supplementary Figure 3. GB formation in NMJs submitted to optogenetic stimulation using red-shifted channelrhodopsin Chrimson (CsChR). **a, b,** Time-lapse images of bouton formation after 3xCsChR stimulation using red light. Neuronal membranes were labeled with UAS-CD4-Tomato (CD4-Tom) under the control of OK6-Gal4 (a MN driver). Scale bar is 2 µm. Arrow indicates where the bouton emerges. **a,** Example of fast bouton formation. **b,** Example of slow bouton formation. **c,** Boxplot (min to max) with the time of bouton formation across experiments. Median is 19 min 08s. All data points are presented. n=6 larvae and 12 boutons. **d,** Representative image of of mVenus-tagged CsChrimson channels expression pattern in MNs. Scale bar is 10 µm. **e,** Images of control and CsChrimson-expressing larvae with and without ATR, after 3xCsChR stimulation. Presynaptic and postsynaptic membranes labeled with antibodies against Horseradish peroxidase (HRP, gray) and Discs-large (Dlg, green), respectively. Ghost boutons (GBs) (arrow) are identified by the lack of Dlg. Scale bar is 10 µm. **f,** Boxplot (min to max) showing GB number after 3xCsChR stimulation. N=41, 53 and 49 NMJs for +>CsChrimson (ATR+), OK6-Gal4 > CsChrimson (ATR+) and OK6-Gal4 > CsChrimson (ATR-), respectively, from 4 biologically independent experiments. Boxplots show 25th–75th percentiles, lines at the median, and whiskers from minimum to maximum. Statistical significance was determined using the two-tailed non-parametric Kruskal-Wallis test, ***p<0.001 (p=0.0001), ****p<0.0001, and ns is not significant. Expression of CsChR in MNs was slightly noxious to the larvae. Expression using NSyb-Gal4 was lethal even in the absence of stimulation of CsChR. Source data provided as a Source Data file.

Supplementary Figure 4

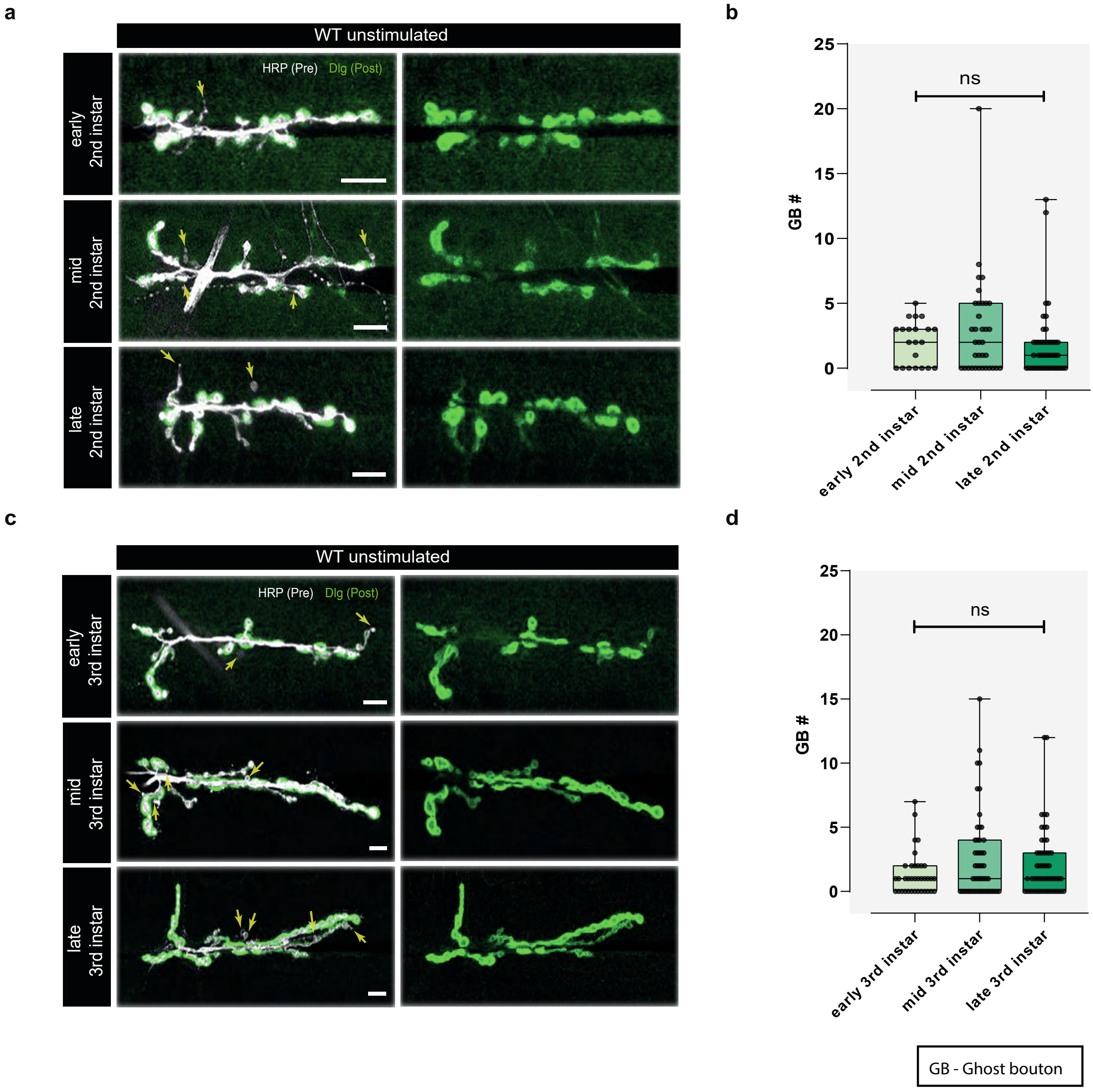

Supplementary Figure 4. GBs are naturally present in NMJs of unstimulated/at rest larvae during larval development. a, Images of unstimulated early, mid, and late 2nd instar larval NMJs. Scale bar in NMJs is 10 µm. **b,** Boxplot (min to max) showing ghost bouton (GB) number at 2nd instar stages. N=21, 35 and 46 NMJs for unstimulated *w1118* early, mid and late 2nd instar larvae, from 2 independent experiments. **c,** Images of unstimulated early, mid, and late 3rd instar larval NMJs. Scale bar in NMJs is 10 µm. **d,** Boxplot (min to max), showing GB number for the 3rd instar stages. N=37, 51 and 56 NMJs for unstimulated *w1118* early, mid and late 3rd instar larvae, from 2 independent experiments. Presynaptic and postsynaptic membranes labeled with antibodies against Horseradish peroxidase (HRP, gray) and Discs-large (Dlg, green), respectively. GBs (arrow) are identified by the lack of Dlg. Boxplots show 25th–75th percentiles, lines at the median, and whiskers from minimum to maximum. Statistical significance was determined using two-tailed parametric analysis of variance (ANOVA), and ns is not significant. Source data provided as a Source Data file.

Supplementary Figure 5

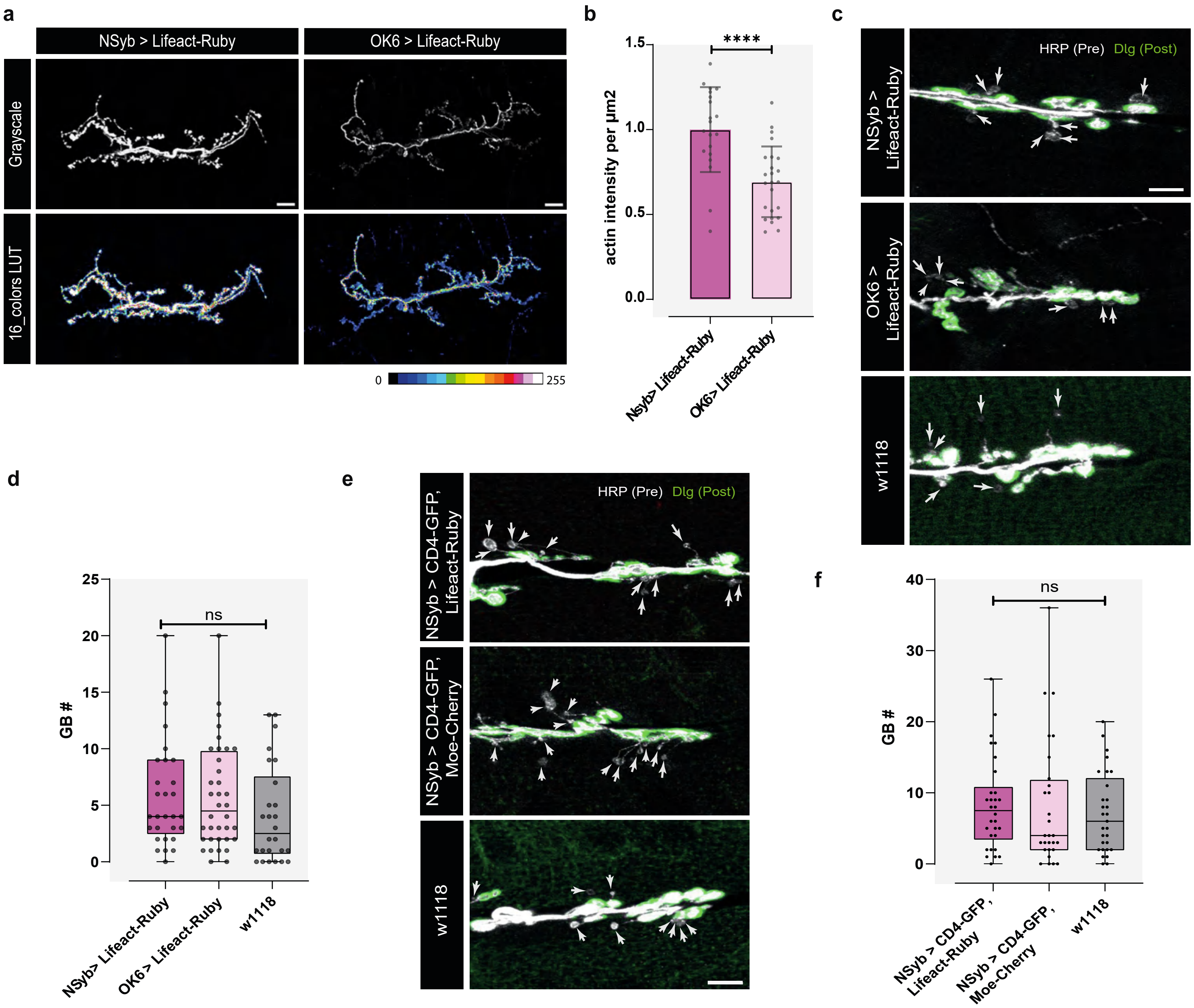

Supplementary Figure 5. Effect of actin levels and different actin reporters on GB formation. a, Images of Lifeact-Ruby (F-actin reporter) expression in the presynaptic terminal under the control of Gal4 drivers with different expression strengths, NSyb (pan-neuronal) and OK6 (MN). The image signal intensity was represented in grayscale (on top) and in 16-LUT (look-up table, bottom) for better visualization. Scale bar is 10 µm. **b,** Scatter dot plot displaying the mean and the error bars represent the S.D., standard deviation, all points represented; data showed that F-actin intensity levels per μm^2 were higher when the reporter was expressed under the control of NSyb-Gal4, compared to OK6-Gal4. N=20 and 24 NMJs for NSyb-Gal4>Lifeact-Ruby and OK6-Gal4>Lifeact-Ruby (actin immunostaining). Statistical significance was determined using a non-parametric Mann-Whitney t-test (two-tailed); ****p <0.0001. **c,** Images of control (WT larvae, w^{1118}) and Lifeact-Ruby expressing larvae under the control of NSyb or OK6 after $3xK^+$ stimulation. Scale bar is 10 µm. **d,** Boxplot (min to max), showing ghost bouton (GB) numbers when Lifeact-Ruby was expressed under the control of NSyb or OK6 Gal4 drivers, compared to WT. All the data points are shown. N=29, 36 and 26 NMJs for NSyb-Gal4>Lifeact-Ruby, OK6-Gal4>Lifeact-Ruby and w^{1118}, respectively, from 2 independent biological experiments. **e,** Images of WT, and transgenic larvae expressing both membrane (CD4-GFP) and F-actin markers (Lifeact-Ruby and Moe-Cherry). Scale bar is 10 µm. **f,** Boxplot (min to max), showing that GB numbers in transgenic larvae compared to WT. n=30, 28 and 29 NMJs for NSyb-Gal4>CD4-GFP,Lifeact-Ruby, NSyb-Gal4>CD4-GFP,Moe-Cherry and w^{1118}, respectively from 2 independent biological experiments. **d, f,** Boxplots show 25th–75th percentiles, lines at the median, and whiskers from minimum to maximum. Statistical significance was determined using a parametric ANOVA test (two-tailed); ns is not significant. Presynaptic and postsynaptic membranes labeled with antibodies against Horseradish peroxidase (HRP, gray) and Discs-large (Dlg, green), respectively. GBs (arrow) are identified by the lack of Dlg. Source data provided as a Source Data file.

Supplementary Figure 6

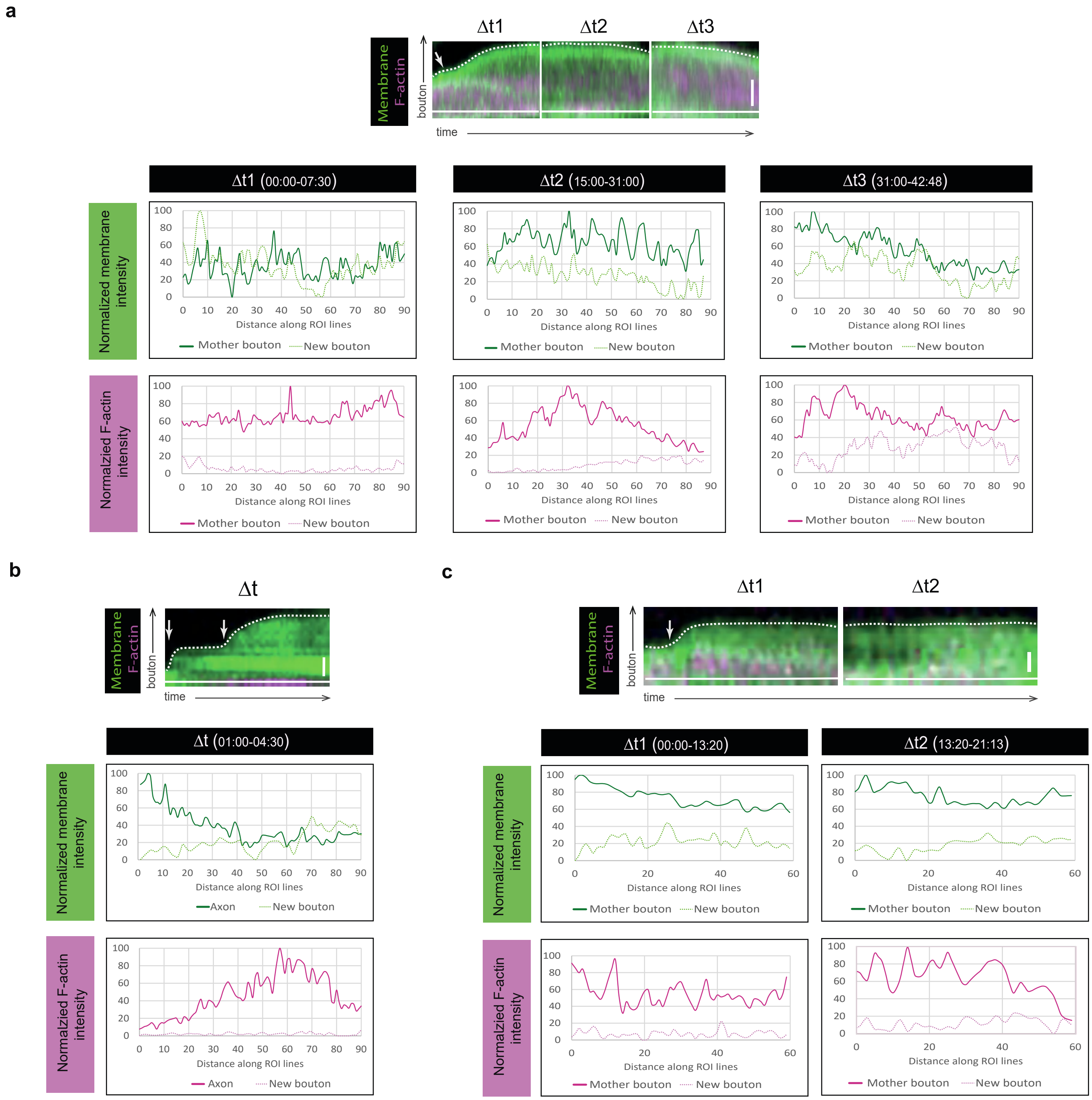

Supplementary Figure 6. Actin dynamics during bouton formation. a-c, Kymographs (top) of distinct ∆t values to highlight the dynamic nature of actin during bouton growth. The neuronal membrane and F-actin were labelled with UAS-CD4-GFP (or CD8-GFP), together with UAS-LifeactRuby under the control of NSyb-Gal4. Scale bars are 1 µm. Arrow indicates where the bouton emerges. Dotted lines along the new bouton border and solid lines along the region underneath the bouton of interested (in a mother bouton or at the axon) and the corresponding plots displaying normalized intensity (below) for the membrane and actin along the base and edge. Min-max normalization of both channels was done for each time interval. **a,** Initially, actin is scarce (∆t1), but is subsequently assembled inside new boutons and accumulates when new bouton size stabilizes (∆t2 and ∆t3). **b,** Actin is absent in new boutons, and an F-actin punctum persists at the base. **c,** Actin begins to assemble after bouton expansion. Source data provided as a Source Data file.

Supplementary Figure 7

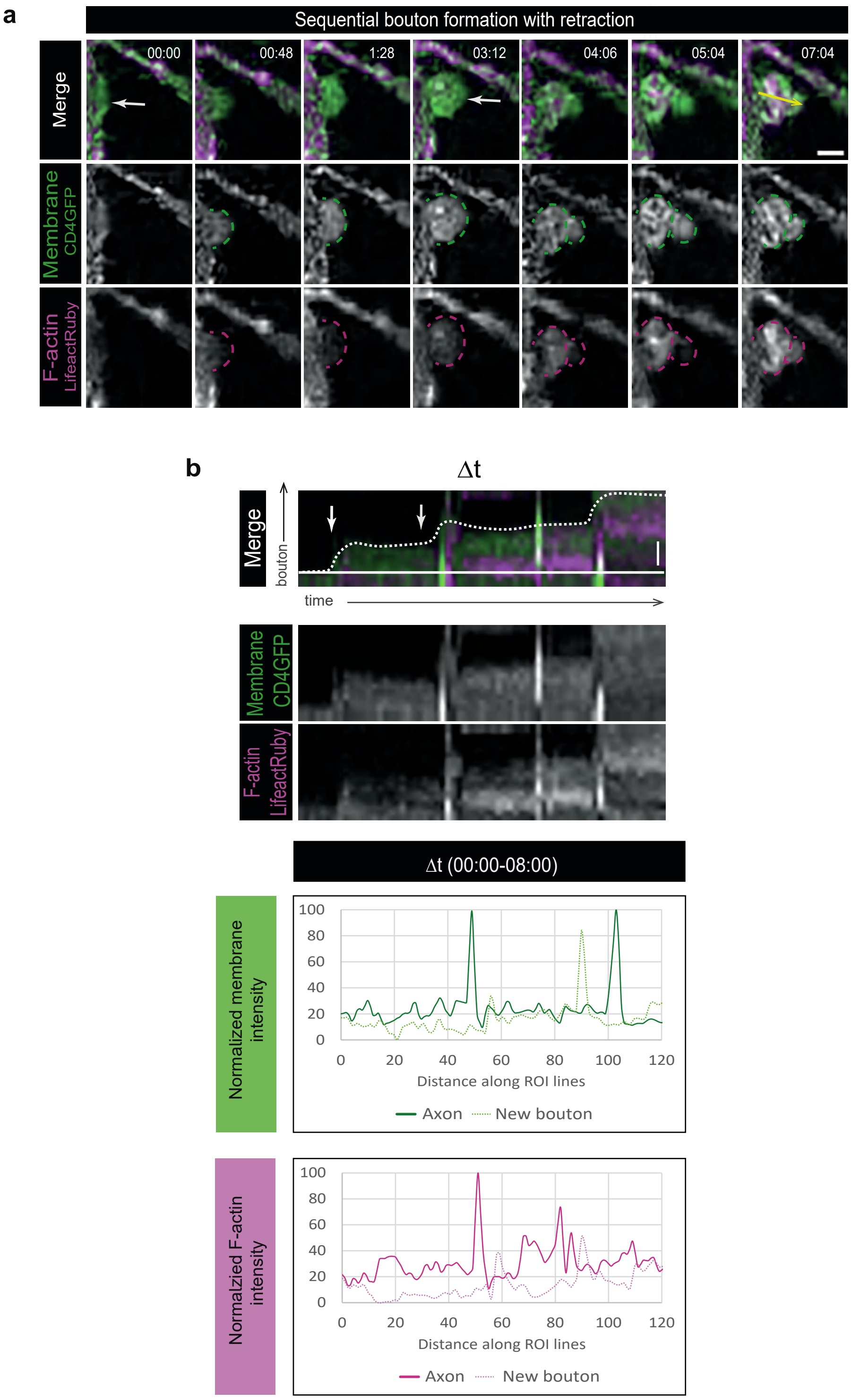

Supplementary Figure 7. Live-imaging example of sequential bouton formation with actin reassembly and reduction in bouton size. **a,** Time-lapse image of actin dynamics in the course of sequential bouton formation with retraction. The neuronal membrane and F-actin were labeled with UAS-CD4-GFP and with UAS-Lifeact-Ruby, both under the control of NSyb-Gal4. Scale bar is 2 µm. White arrow indicates where the bouton emerges. Yellow arrow indicates ROI used for kymograph generation. **b,** Kymograph from a) showing that in the beginning actin is weak and accumulates when new boutons stop growing/retract Scale bar is 1 µm. We represent dotted lines in the new bouton border and solid lines along the region underneath the bouton of interested (in a mother bouton or at the axon) and the corresponding normalized intensity (min-max normalization) values for the membrane and F-actin are plotted below. Source data provided as a Source Data file.

Supplementary Figure 8

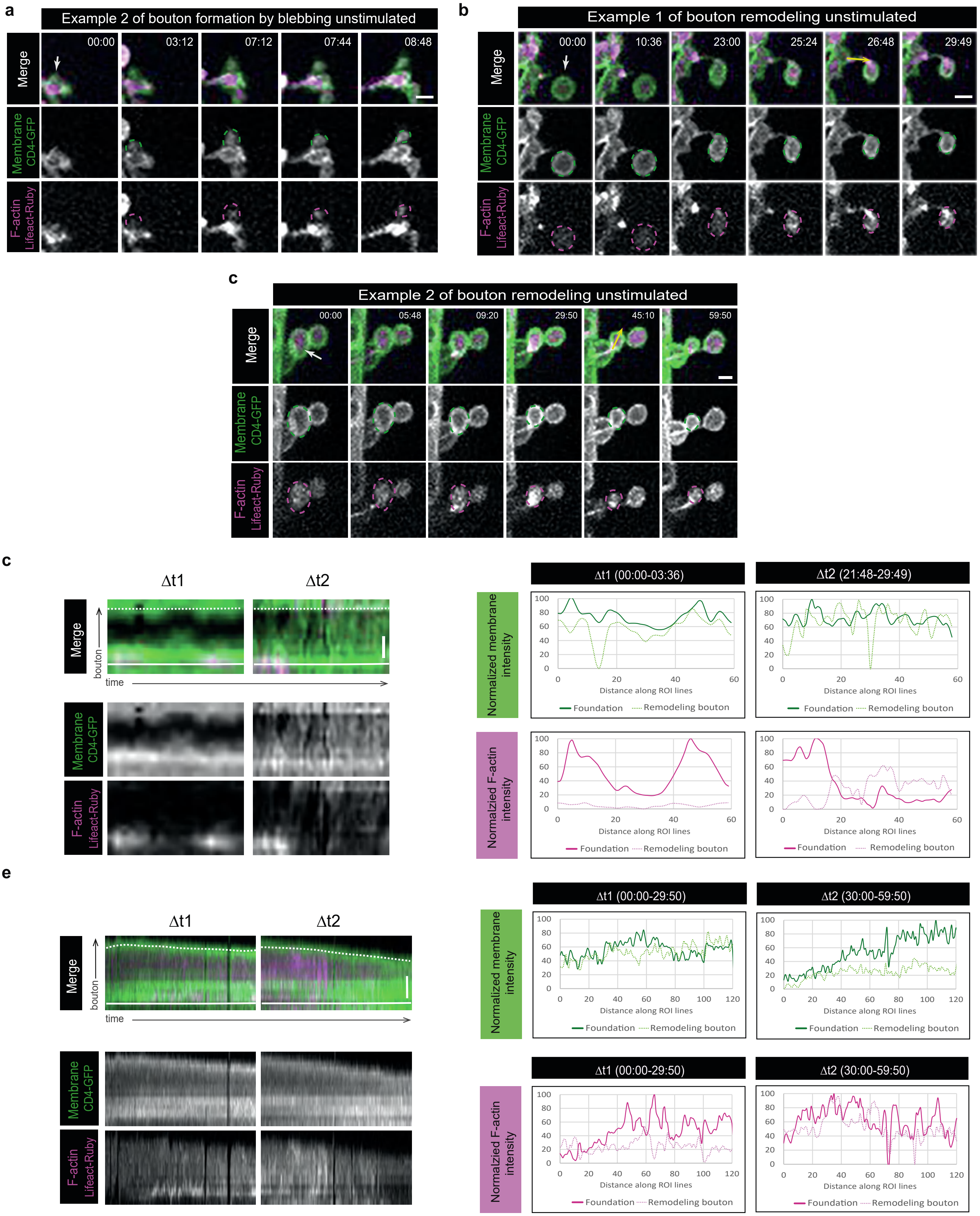

Supplementary Figure 8. Live examples of bouton formation and remodeling without stimulation. a, Time-lapse images of bouton formation without stimulation showing the bleb life phases. Representative images were obtained with HL3.1 including 0.1 mM Ca^{2+} (Example 2 in addition to Fig. 2h). **b,c,** Time-lapse images of two examples of bouton remodeling (shrinkage) in unstimulated conditions associated with clear actin accumulation. The neuronal membrane and F-actin were labeled with UAS-CD4-GFP and UAS-Lifeact-Ruby, both under the control of NSyb-Gal4. Scale bars are 2 µm. Arrow indicates the location of bouton addition (b) or remodeling (c). **d,e,** Kymographs of b and d. Scale bars are 1 µm. Kymographs from distinct Δt's (top) showing actin recruitment to boutons that were remodeled. Dotted lines along remodeling boutons and solid lines indicating bouton's foundation (place from where bouton emerges); we show the corresponding plots displaying normalized intensity values (below) for the membrane and actin. Min-max normalization of both channels was done for each time interval. Source data provided as a Source Data file.

Supplementary Figure 9

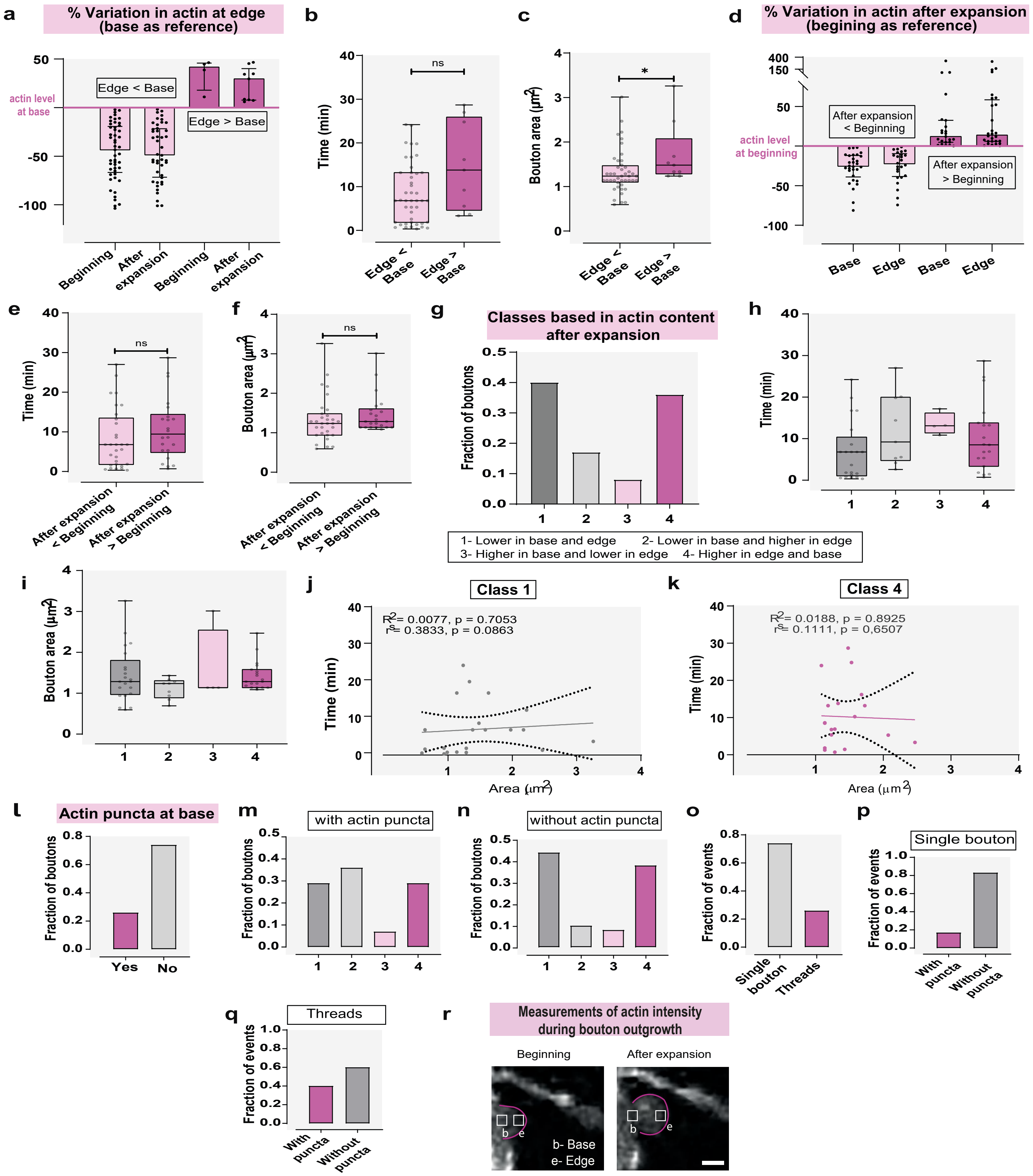

Supplementary Figure 9. Actin content/changes in new bouton edge and/or base and analysis of bouton formation dynamics. a, Scatter dot plots (median with interquartile range) showing % variation in F-actin intensity at growing bouton's edge compared to their base (measured at beginning and after expansion) to assess the amount of F-actin recruited to boutons. **b, c**, Boxplot (min to max) showing bouton formation times (b) and area (c) when F-actin intensity at edge was lower than base (measured after expansion). **c,** We observed a marginal increase in bouton area when F-actin intensity at bouton's edge was higher than their base. Line represents median. **d,** Scatter dot plots (median with interquartile range) showing % variation in F-actin intensity in growing bouton's bases or edges. For each location, we compared values obtained after expansion with the ones at beginning to assess F-actin flow throughout bouton outgrowth. **e, f**, Boxplot (min to max) showing time of bouton formation (e) and bouton area (f) in the bouton's edges that formed with lower or higher F-actin (relative to beginning). **b,c,e,f,** Statistical significance was determined using non-parametric Mann-Whitney test (two-tailed); *$p<0.05$ ($p= 0.0196$), ns is not significant. **g,** Plot with fraction of boutons that after expansion show actin: 1- lower in edge and base; 2- lower in edge and higher in base; 3- higher in edge and lower in base; 4 – higher in edge and base. **h,** Boxplot (min to max) showing times of bouton formation in classes 1-4. **i,** Boxplot (min to max) showing the bouton areas in classes 1-4. **h,i,** No trend in formation time was observed. Line represents the median. All data points are presented. **j,k,** Linear regression plots between time and area for classes 1 **(j)** and 4 **(k)**. 95% confidence bands of the best-fit line are indicated by dotted lines. R^2, Spearman correlation (r^s), and respective p-values, are shown in the graphs. No linear relationship was found. **l,** Plot with fraction of boutons with actin puncta at the base. **m,n,** Plot with fraction of boutons of classes 1-4 with (m) or without (n) actin puncta at the base. **o,** Plot with fraction of events that lead single or threads of boutons. **p, q,** Plots with fraction of single boutons (p) or threads with boutons (q) with or without actin puncta. **r,** Schematic for actin quantification. Scale bar is 2μm. F-actin fluorescence intensity was measured in each bouton edge (e) and at the base (b) at the initial frame (beginning) and at maximal size (when expansion stopped). ROI was a 1x1 μm square. Data represents 14 larvae and 53 boutons. Boxplots show 25th–75th percentiles, lines are at the median, and whiskers from minimum to maximum. Source data provided as a Source Data file.

Supplementary Figure 10

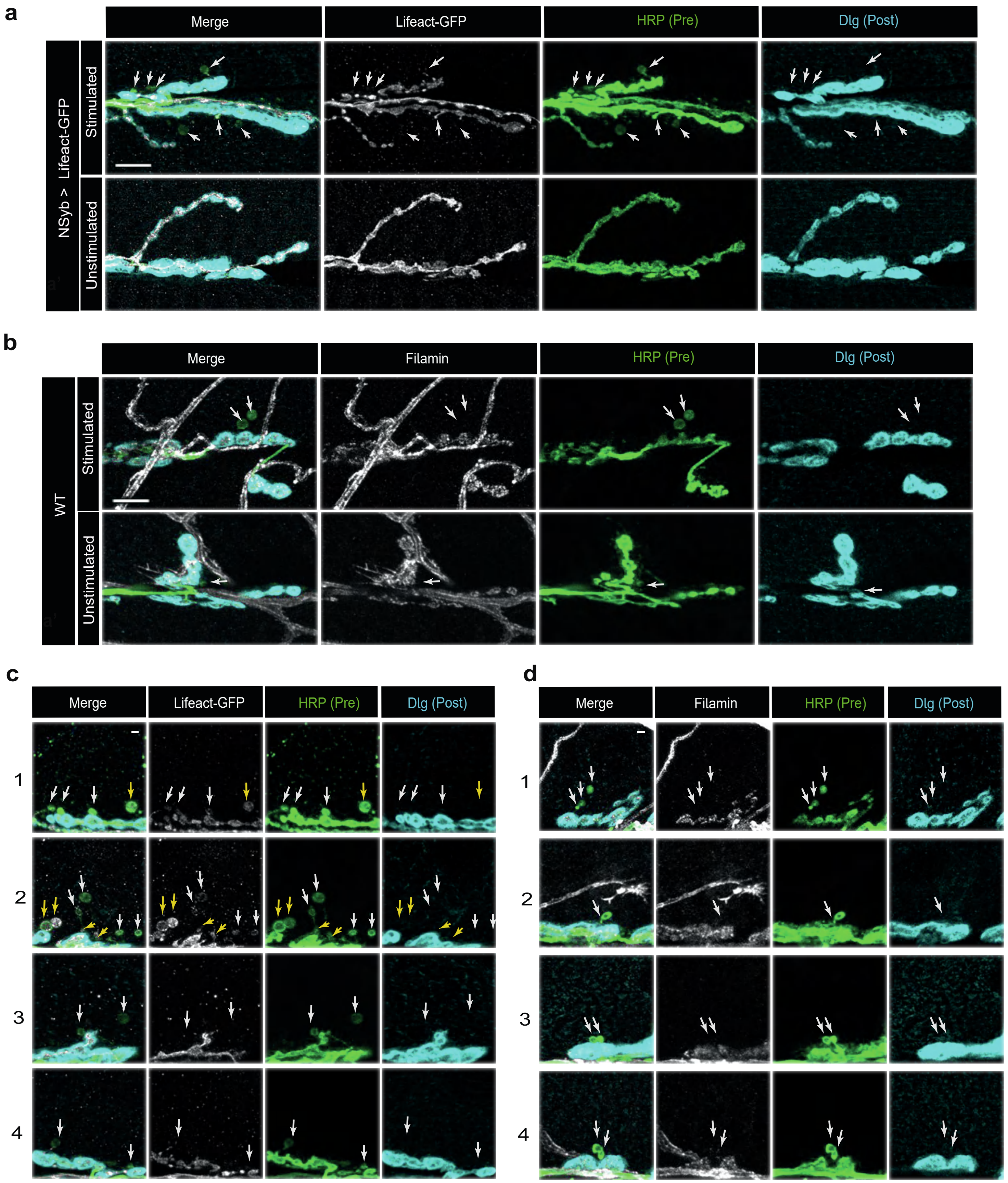

Supplementary Figure 10. F-actin and filamin localization at the NMJ before and after high-K$^+$ stimulation. **a,** Representative examples of 3rd instar larvae expressing UAS-Lifeact-GFP under the control of NSyb-Gal4, labeled with anti-GFP antibody to detect F-actin distribution in neurons. Scale bar is 10 µm. **b,** Representative examples of WT (*W*^{1118}) larvae labeled with anti-filamin antibody (anti-Cheerio antibody, green) (Lee & Schwartz, 2016) at rest (unstimulated, bottom panels) and post high K$^+$ stimulation (top panels). The presynaptic and postsynaptic membranes were labeled with antibodies for Horseradish peroxidase (HRP,green) and Discs-large (Dlg, cyan), respectively. Ghost boutons (GBs) (arrow) are identified by the lack of Dlg. Scale bar is 10 µm. **c,d** Examples of F-actin (c) or filamin (d) localization in GBs. **c,** Examples of GBs without actin or with low amounts of actin (white arrows), and of actin-rich GBs (yellow arrows), reminiscent of the described bleb stages. Scale bar is 2 µm. **d**, Despite present in MNs terminal boutons, filamin is not recruited to newly formed boutons (white arrows), suggesting that the actin structure is weaker in the newly formed boutons. Scale bar is 2 µm. **a, c,** data is representative of Stim: 15 NMJs, 6 larvae; Unstim: 11 NMJs, 6 larvae, from 2 independent experiments. **b,d,** data is representative of Stim: 30 NMJs, larvae; Unstim: 15 NMJs, 6 larvae, from 2 independent experiments.

Supplementary Figure 11

a

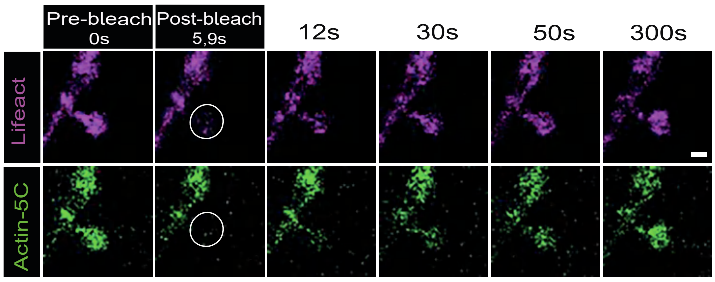

b

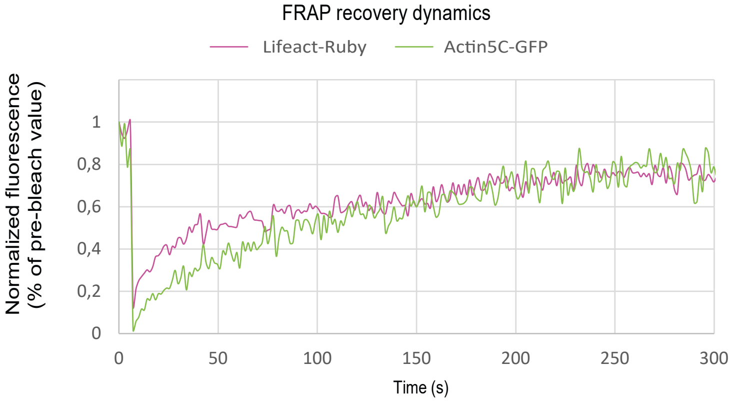

c

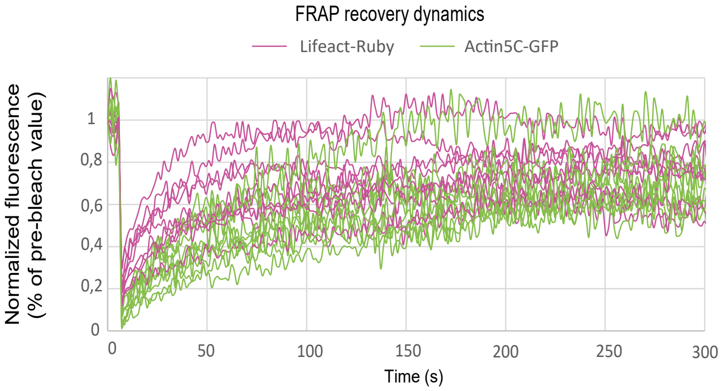

Supplementary Figure 11. FRAP experiments on F-actin reporters in synaptic boutons. a, Representative images of a bouton co-expressing Actin5C-GFP (green) and Lifeact-Ruby (magenta) (under control of NSyb-Gal4), which were photobleached and allowed to recover for 300s. The white circle indicates the ROI for photobleaching. Scale bar is 2 μm. **b,** Representative normalized FRAP curves (to prebleach intensity) obtained from plot against time. **c,** Normalized FRAP curves (to prebleach intensity) obtained from each bouton analysis overlay. We observed comparable recovery dynamics after bleaching for Actin5C- and Lifeact based reporters. n= 5 larvae and 9 boutons, from 2 independent experiments. Source data provided as a Source Data file.

Supplementary Figure 12

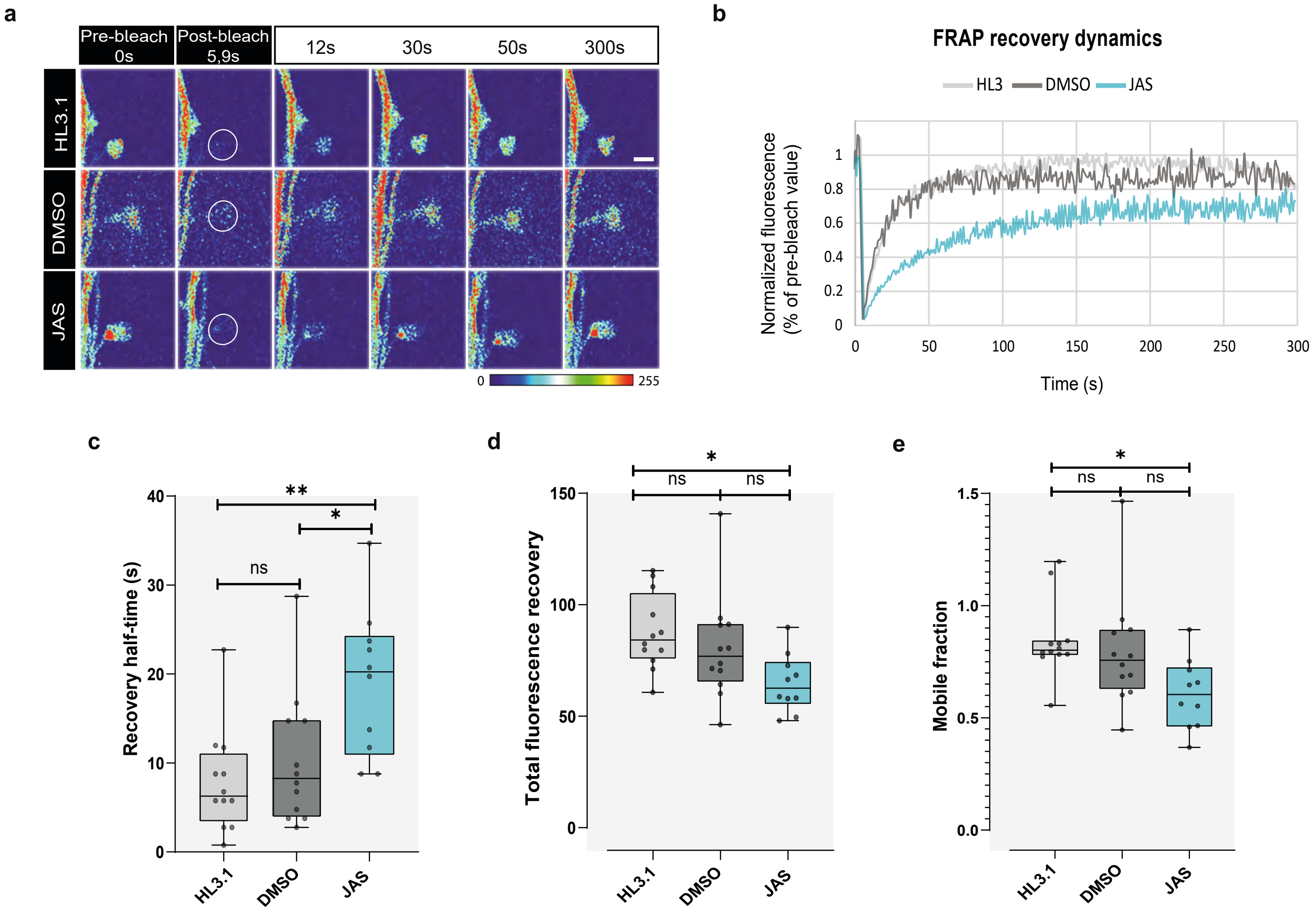

Supplementary Figure 12. Effects of JAS treatment on presynaptic actin dynamics. a, Time-lapse images showing Lifeact-GFP expression in the presynaptic terminals during FRAP. Photobleaching of F-actin probe in synaptic boutons to examine actin dynamics after Jasplakinolide (JAS) treatment, compared to controls (HL3.1 and DMSO). White circles indicate photobleaching ROI. Scale bar is 2 µm. **b,** Normalized FRAP curves from c) plotted against time, showing fluorescence recovery. **c,** Boxplot (min to max) showing recovery half-time (s). Lines represent median. Statistical significance determined using parametric analysis of variance (ANOVA); p-value HL3-JAS= 0.0029, DMSO-JAS= 0.0206; **d,e,** FRAP analysis indicating F-actin stabilization with JAS. **d,** Box-plot (min to max) showed fluorescence recovery after bleaching for JAS and controls (HL3.1 and DMSO). Fluorescence recovery after photobleaching was decrease after JAS treatment. P-value HL3-JAS= 0.0192. **e,** Boxplot (min to max) showing the mobile fraction after photobleaching. JAS application also decreased the mobile fraction. P-value HL3-JAS= 0.0183; HL3: n=12 NMJs/10 larvae; DMSO: n=12NMJs/12larvae; JAS: n=10NMJs/10 larvae. from 3 independent experiments. Boxplots show 25th–75th percentiles, lines at the median, and whiskers from minimum to maximum. Statistical significance was determined using the parametric ANOVA test; **p<0.01, *p<0.05, ns not significant. Source data provided as a Source Data file.

Supplementary Figure 13

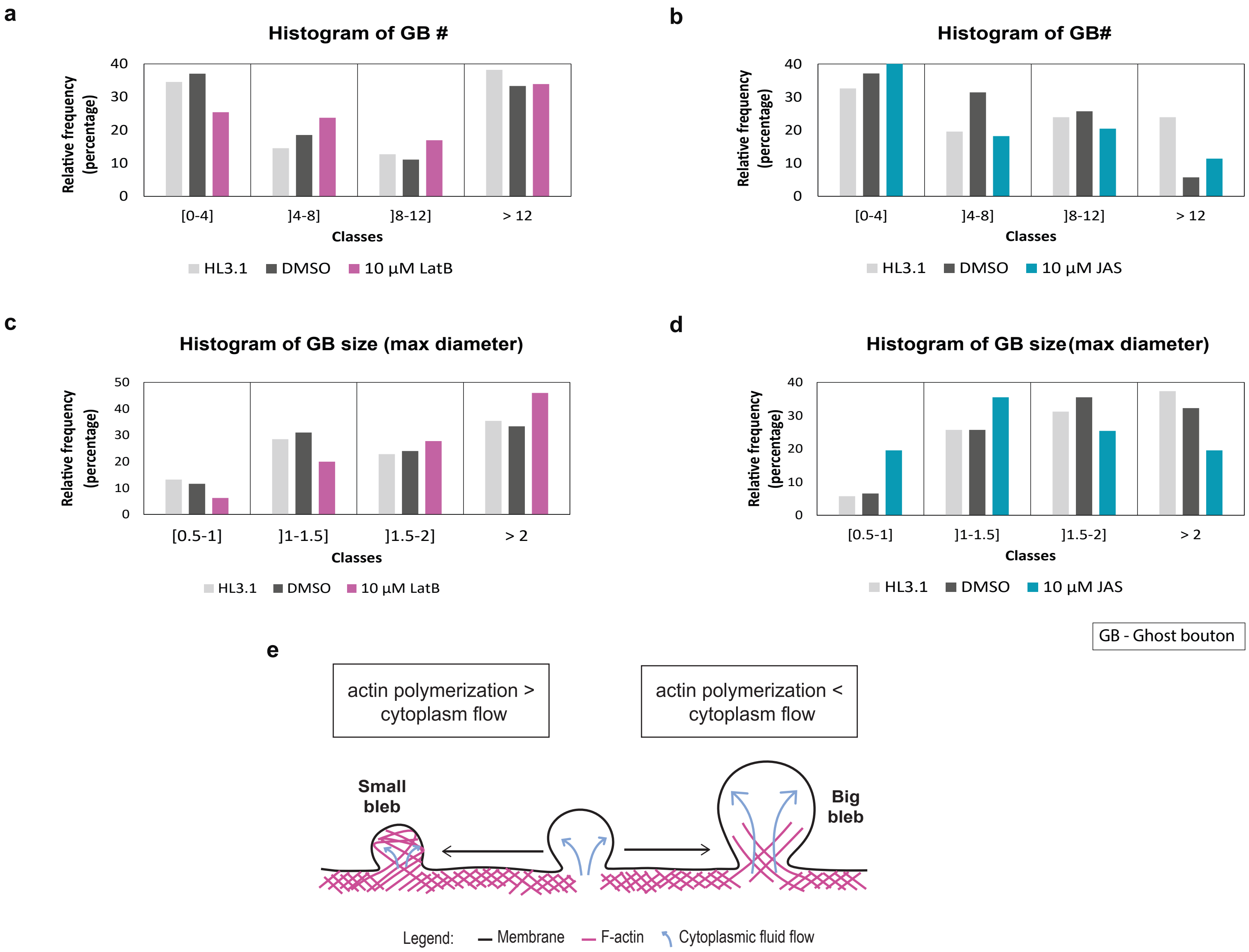

Supplementary Figure 13. GB number and size distribution plots after manipulation of actin dynamics and schematic of the hypothesis of actin-dependent regulation of bouton size. **a,** Histogram showing percentage of Ghost boutons (GB) numbers in each class for WT NMJs treated with HL3.1, DMSO (solvent control), or 10 µM Latrunculin B (LatB). n=59, 56 and 55 NMJs for HL3, DMSO and LatB, respectively from 3 independent experiments. **b,** Histogram showing percentage of GBs numbers in each class for WT NMJs treated with HL3.1, DMSO (solvent control), or 10 µM Jasplakinolide (JAS). n=44, 35 and 46 NMJs for HL3, DMSO and JAS, respectively from 3 independent experiments. **c,** Histogram showing percentage of GB sizes (maximal diameter) in each class after LatB application. N=779, 629 and 779 GBs for HL3, DMSO and LatB, respectively, and 3 biologically independent experiments. **d,** Histogram showing percentage of GB sizes (maximal diameter) after JAS application. n=471, 214 and 307 GBs for HL3, DMSO and JAS, respectively. **e,** Schematics of the contribution of actin polymerization to bleb size regulation. Bleb size is controlled by a balance between cytoplasmic flow, which drives expansion, and actin polymerization, which stops growth. Source data provided as a Source Data file.

Supplementary Figure 14

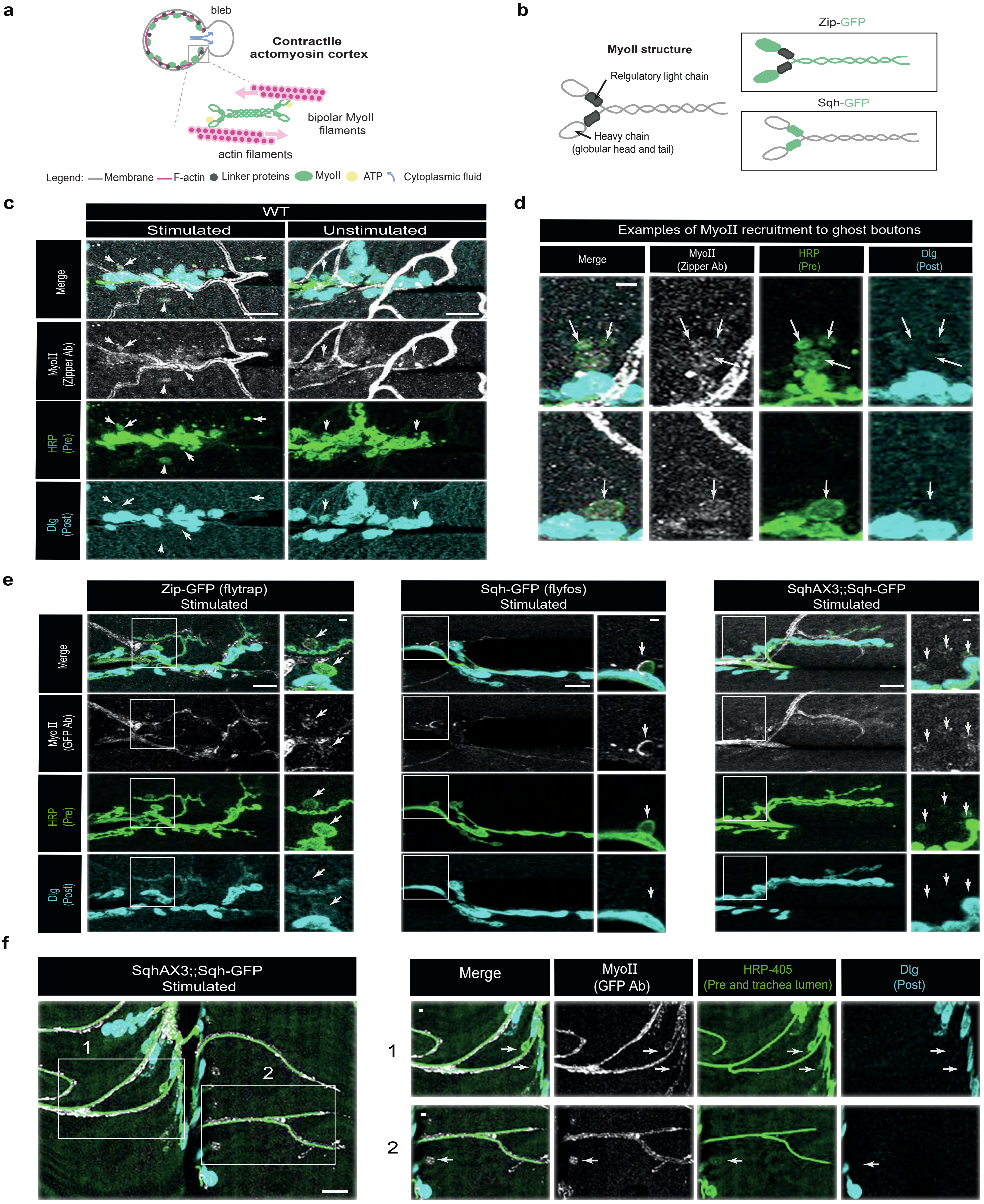

Supplementary Figure 14. MyoII localization at the NMJ after high-K$^+$ stimulation. **a,** Schematics of the structure of the actomyosin cortex described in cells and of non-muscle myosin-II (MyoII) motor complex. **b,** Each molecule of MyoII is composed of two heavy chains (Zipper - Zip) and two regulatory light chains (Spaghetti squash - Sqh). **c,** Antibody staining for MyoII (Zipper Ab, gray) in WT (w^{1118}) to observe endogenous distribution at the NMJ. Stimulated NMJ (left). Unstimulated NMJ (right). Scale bars are 10 µm. MyoII is present in the presynaptic terminal, sometimes outlining the boutons, and is abundant in the muscle and trachea. N= 6 larvae/28 NMJs, and 3 biologically independent experiments. **d**, post-stimulation MyoII is recruited to ghost boutons (GBs) with visible accumulations at the base and edge. Scale bar is 2 µm. **e,** GFP protein traps of Sqh or Zip (schematized in b) corroborate the myosin cellular distribution observed by antibody staining (Fig.3). GFP-tagged Sqh in an Sqh-null background (Sqh^{AX3}) also showed normal MyoII localization at the NMJ. Scale bars are 10 µm. **f,** MyoII localizes to trachea, as it co-localizes with autofluorescent (405 nm) tracheal lumen. Data in this figure are representative of one experiment for Zip-GFP and Sqh-GFP lines (N=6 larvae, 10 NMJs per genotype) and at least 3 biologically independent experiments for Sqh^{AX3};;Sqh-GFP (N=6 larvae, 39 NMJs). Scale bar is 10 µm. **c-f,** Presynaptic and postsynaptic membranes labeled with antibodies for Horseradish peroxidase (HRP,green) and Discs-large (Dlg, cyan), respectively. GBs (white arrow) are identified by the lack of Dlg. Scale bar, 10 µM.

Supplementary Figure 15

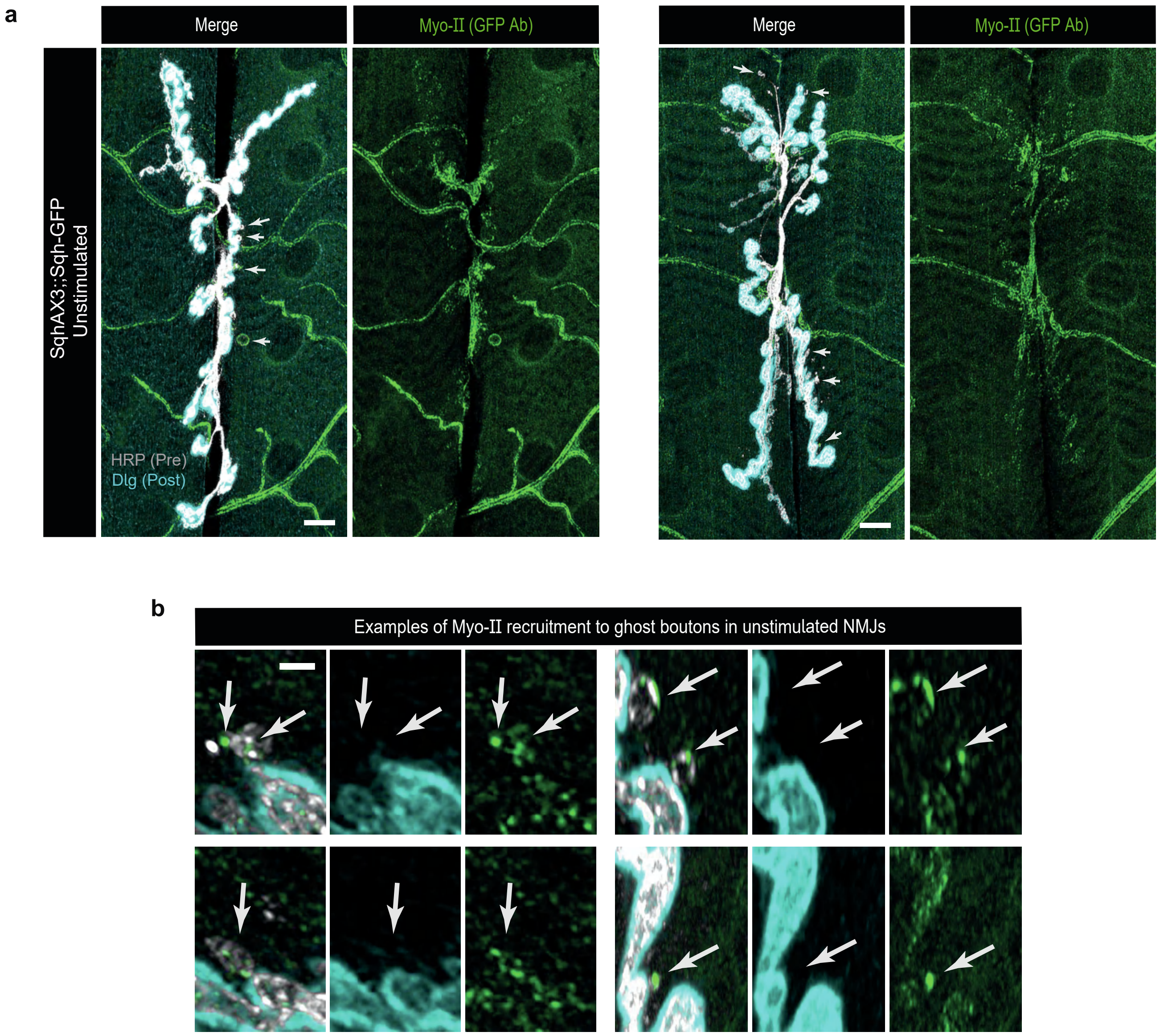

Supplementary Figure 15. MyoII localization in GBs of unstimulated NMJs. a, Representative images of non-muscle myosin-II (MyoII) (green) distribution at NMJs observed without simulation. Although MyoII is highly expressed in muscle and trachea, it is clearly visible in the presynaptic terminal and delineating boutons. MyoII was visualized by antibody staining against GFP, in a GFP-tagged Spaghetti squash (Sqh, regulatory light chain) present in a Sqh-null background (Sqh^{AX3}). The presynaptic and postsynaptic membranes were labeled with antibodies for Horseradish peroxidase (HRP, gray) and Discs-large (Dlg, cyan), respectively. Ghost boutons (GBs) are identified by the lack of Dlg. N=18 NMJs, 10 larvae, 1 experiment. Scale bar, 10 μM. **b,** Similar to what was observed post-stimulation, MyoII accumulated in GBs at rest conditions (arrows). Scale bar is 2 μm.

Supplementary Figure 16

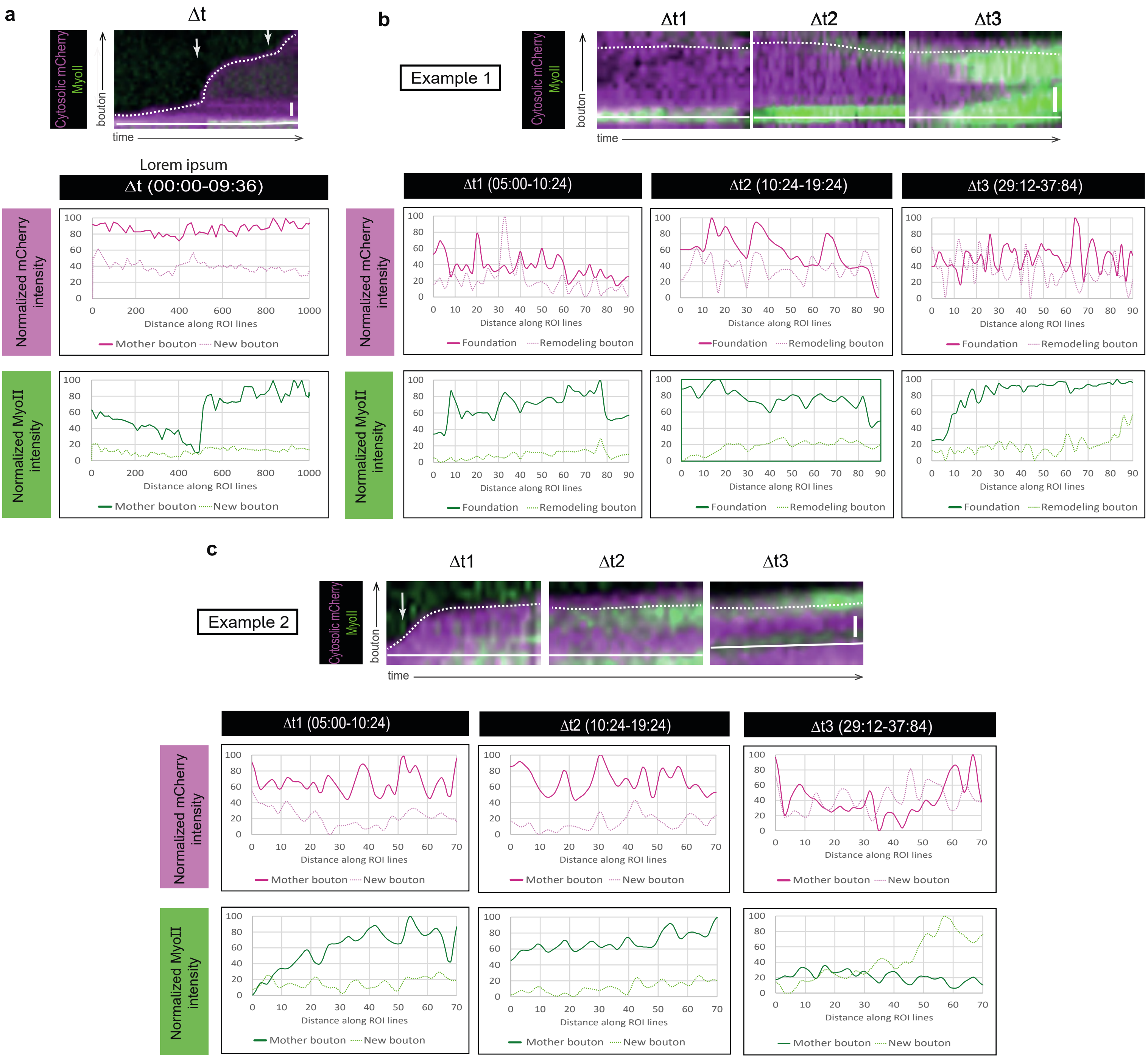

Supplementary Figure 16. MyoII dynamics during bouton formation. Kymographs (top) of distinct Δt values to highlight the dynamic nature of MyoII during bouton formation and remodeling. Bouton growth was visualized with cytosolic LexAop-mCherry under the control of the LexA driver DvGlut, and to label non-muscle myosin-II (MyoII) we used GFP-tagged Spaghetti squash - Sqh (regulatory light chain) in a Sqh-null background (Sqh^{AX3}). Arrow indicates where the bouton emerges. Dotted lines along new bouton border (or remodeling bouton) and solid lines along region underneath the bouton of interest (in a mother bouton or at the axon) and corresponding plots displaying normalized intensity values (below) for the mCherry and MyoII. Min-max normalization of both channels was done for each time interval. **a,** MyoII persists at the base during bouton growth. **b,** MyoII clustered at the bouton's border, while bouton size was reduced (Δt2 and Δt3). **c,** Small bouton forming at the edge (arrow) of the parental bouton (Δt1) and MyoII clustering as the bouton became smaller (Δt2 e Δt3). **b**, Kymograph and intensity profiles relative to example of MyoII clustering represented in Fig 4e as 1 (in yellow). **c**, Kymograph and intensity profiles relative to example 2 of MyoII clustering shown in Fig. 4e as 2 (in yellow). Scale bars in kymographs are 1 µm. Representative of n= 9 NMJs, 9 larvae and 92 boutons; 5 independent experiments. Source data provided as a Source Data file.

Supplementary Figure 17

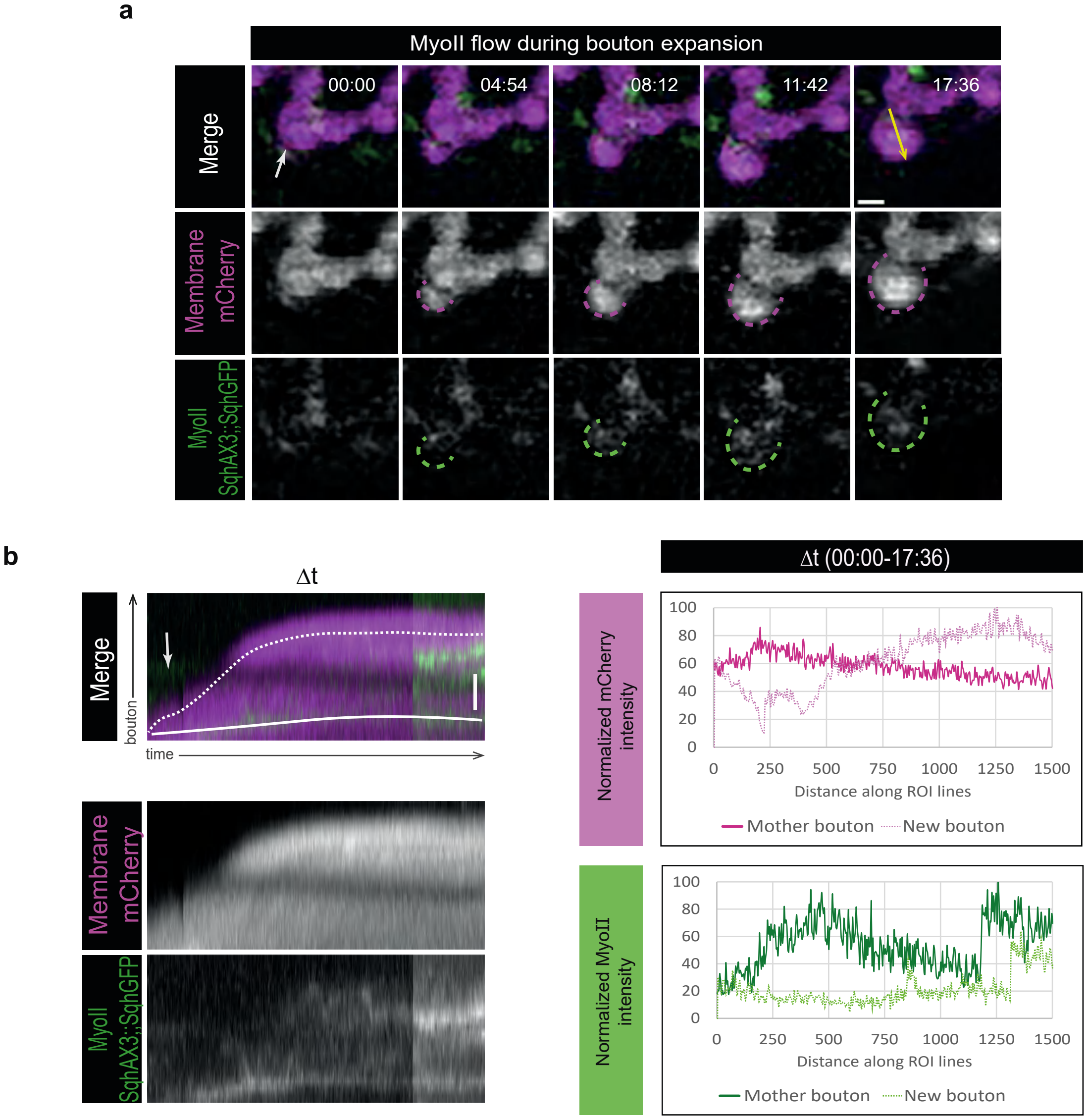

Supplementary Figure 17. Live imaging example of MyoII flow during bouton growth. **a,** Time-lapse image of an example of non-muscle myosin-II (MyoII) flow inside the bouton during its expansion. To follow bouton growth, we used cytosolic monomeric Cherry (mCherry) under the control of LexA driver DvGlut and labeled MyoII using GFP-tagged Spaghetti squash-Sqh (regulatory light chain) in a Sqh-null background (Sqh^{AX3}). Scale bar, 2 µm. Arrow indicates where the bouton emerges. **b,** Kymograph from example in **a)** showing MyoII dynamics for a particular Δt. Tracing lines are represented along the border of the new bouton (dotted lines) and along the region underneath the bouton of interest (in a mother bouton); the corresponding normalized intensity values (min-max normalization) for mCherry and MyoII channels are plotted on the right. Scale bar in kymographs is 1 µm. Representative of n= 9 NMJs, 9 larvae and 92 boutons; 5 independent experiments. Source data provided as a Source Data file.

Supplementary Figure 18

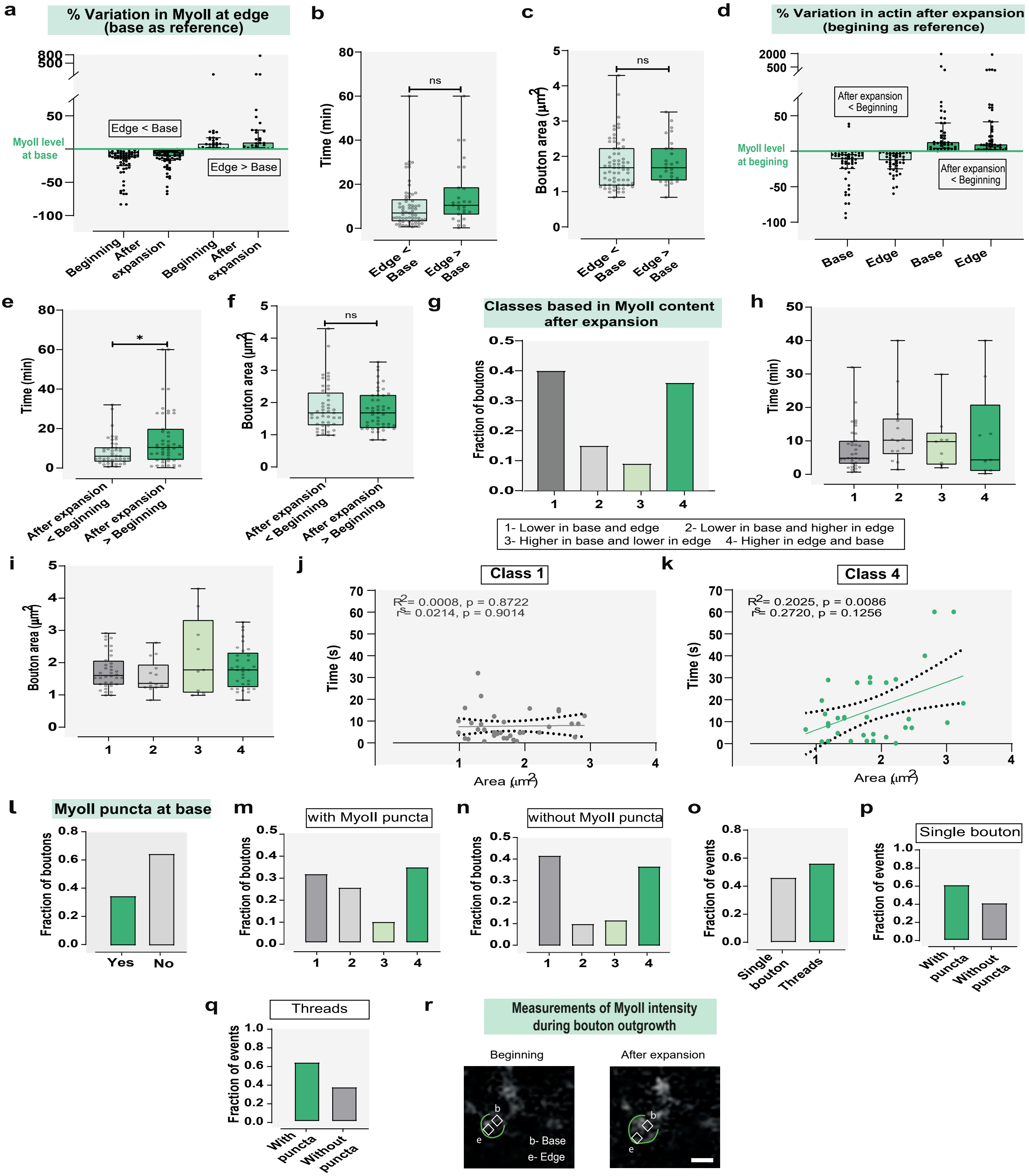

Supplementary Figure 18. Analysis of MyoII content/changes in new bouton edge and/or base and analysis of bouton formation dynamics. **a,** Scatter dot plots (median with interquartile range) showing % variation in non-muscle myosin-II (MyoII) intensity at growing bouton's edge compared to their base (measured at beginning and after expansion) to assess the amount of MyoII recruited to boutons. **b,c,** Boxplots (min to max) showing bouton formation time (b) and bouton area (c) when MyoII intensity at edge was lower or higher than base (measured after expansion). Line represents median. **d,** Scatter dot plots (median with interquartile range) showing % variation in bouton's base or edge MyoII intensity. For each location, we compared values obtained after expansion with the ones at beginning to assess MyoII flow throughout bouton outgrowth. **a,d,** The YY-axis was segmented into discrete intervals to allow range visualization of the data. **e, f,** Boxplot (min to max) showing bouton formation time (e) and area (f) in bouton's edges that formed with lower or higher MyoII (relative to beginning). **e**, Bouton formation was faster when MyoII levels were lower at the bouton edge. Line represents median. P-value=0.0302. **b,c,e,f,** Statistical significance was determined using non-parametric Mann-Whitney test (two-tailed); *p<0.05, ns is not significant. **g,** Plot with fraction of boutons that at maximum size show MyoII: 1- lower in edge and base; 2- lower in edge and higher in base; 3- higher in edge and lower in base; 4 – higher in edge and base. **h,** Boxplot (min to max) showing bouton formation times in classes 1-4. Line represents median. **i,** Boxplot (min to max) showing bouton areas in classes 1-4. Line represents median**. j,k,** Linear regression plots between time and area for classes 1 (j) and 4 (k). 95% confidence bands of the best-fit line are indicated by dotted lines. R^2, Spearman correlation (r^s), and respective p-values, are shown in the graphs. Class 4 showed a weak linear relationship between time and area. **l,** Plot with fraction of boutons with MyoII puncta at the base. **m,n,** Plot with fraction of boutons of classes 1-4 with (m) or without (n) myosin puncta at the base. o, Plot with fraction of events leading to formation of single or threads of boutons. **p,q,** Plots with fraction of single boutons (p) or bouton threads (q) with or without MyoII puncta. Boxplots show 25th–75th percentiles, lines at the median, and whiskers from minimum to maximum. **r,** Schematic for MyoII quantification. Scale bar is 2 µm. MyoII fluorescence intensity was measured at bouton edge and base at the initial frame (beginning) and maximal size (when expansion stopped). ROI was a 1x1 µm square. Representative of n= 9 NMJs, 9 larvae and 92 boutons; 5 independent experiments. Source data provided as a Source Data file.

Supplementary Figure 19

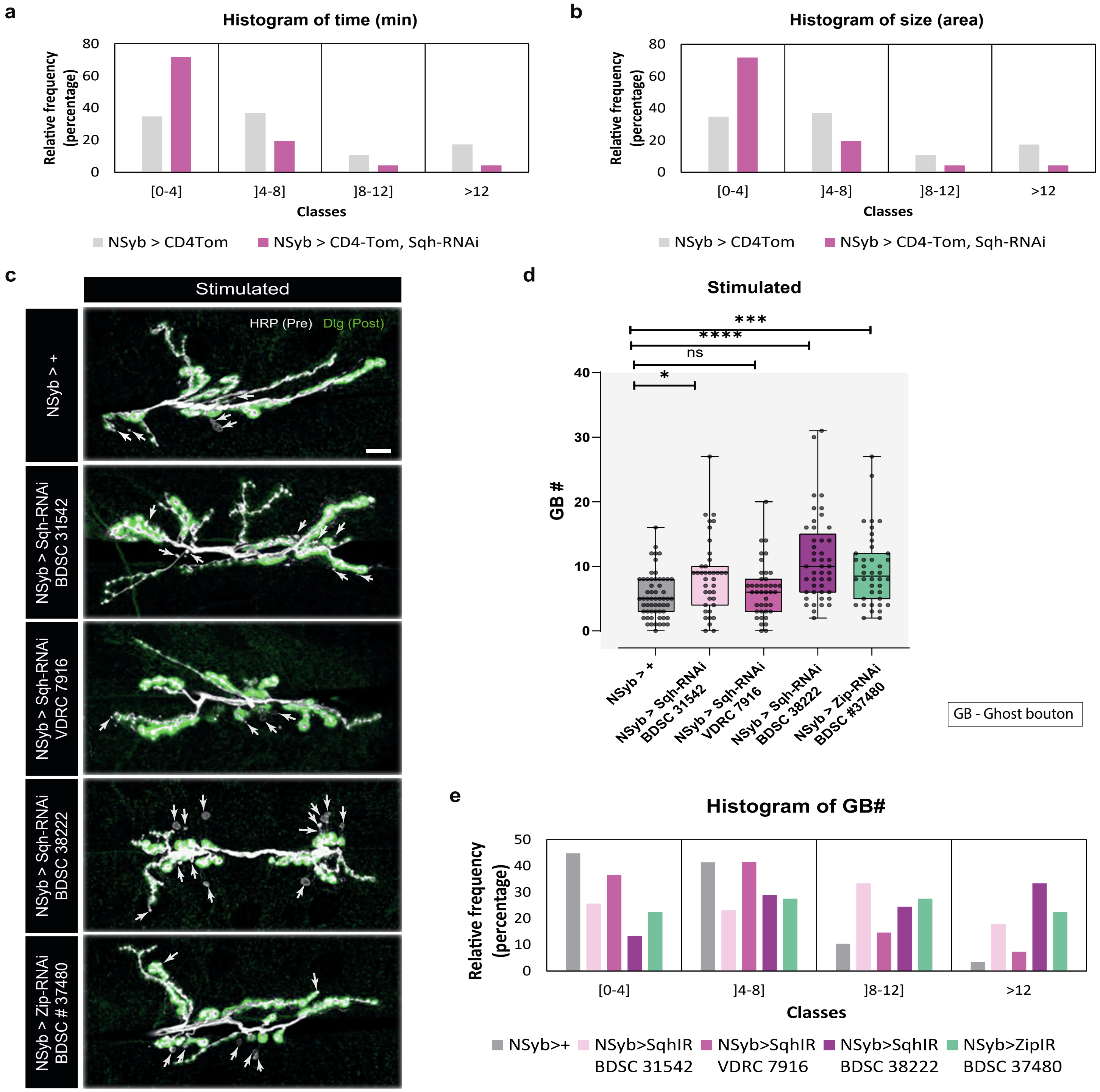

Supplementary Figure 19. Effect of reducing MyoII on activity-dependent plasticity. a,b, Histograms of percentage for times of bouton formation (a) and bouton area (b) in each class for NSyb-Gal4>CD4-Tomato (CD4-Tom),+ and in NSyb-Gal4>CD4-Tom,Sqh-RNAi. N=13 larvae and 46 boutons (for control), and n = 5 larvae and 46 boutons (for Sqh-RNAi). **c,** Images of control animals and animals expressing additional RNAi lines targeting the non-muscle myosin-II (MyoII) light (Spaghetti squash, Sqh) or heavy chain (Zipper, Zip) chain. We used NSyb-Gal4 (pan-neuronal driver) to express the MyoII RNAis in the motor neurons (MNs) (in addition to the pre-validated RNAi lines by Nie *et al.* (2014), which are displayed in the main Fig. 5). The presynaptic and postsynaptic membranes were labeled with antibodies for Horseradish peroxidase (HRP, gray) and Discs-large (Dlg, green), respectively. Ghost boutons (GBs) (arrow) are identified by the lack of Dlg. Scale bar, 10 μm. **d,** Boxplot (min to max) showing GB number for all the MyoII RNAi lines tested. We found that neuronal expression of all but one MyoII RNAi lines tested led to increased GB numbers after acute stimulation, compared to the control. Line is at median. All data points are presented. **e,** Histogram showing the percentage of GBs in each class for the stimulated NMJs. **d,e**, n = 58 (NSyb-Gal4>+), 39 (NSyb-Gal4>Sqh-RNAi #2), 41 (NSyb-Gal4>Sqh-RNAi #3), and 45 (NSyb-Gal4>Sqh-RNAi #4), 40(NSyb-Gal4>Zip-RNAi #2). Boxplots show 25th–75th percentiles, lines at the median, and whiskers from minimum to maximum. Statistical significance was determined with parametric ANOVA test (two-tailed);****p<0.0001, ***p<0.001, *p<0.05, and ns is not significant. P-value Control-Sqh-RNAi#2= 0.0359, P-value Control-Sqh-RNAi#4<0.0001, P-value Control-Zip-RNAi#2= 0.0012. Source data provided as a Source Data file.

Supplementary Figure 20

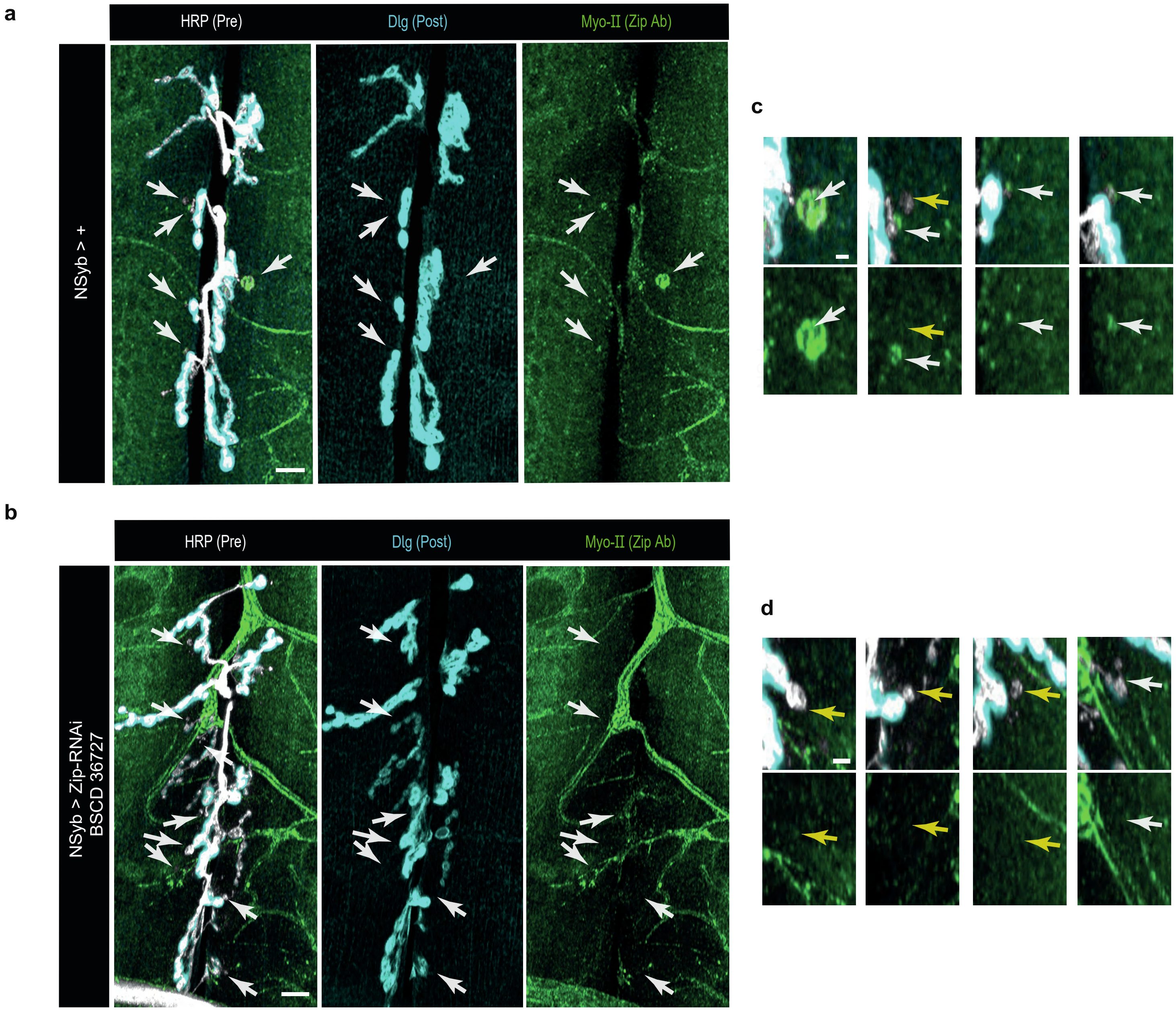

Supplementary Figure 20. MyoII localization in GBs in control and MyoII K/D NMJs after high-K$^+$ stimulation. **a,b,** Representative images of non-muscle myosin-II, MyoII antibody staining (Zipper antibody – Zip Ab, green) in control (a) and Zip-RNAi (b) NMJs after stimulation. We used NSyb-Gal4 (a pan-neuronal driver) to express Zip-RNAi in motor neurons. The presynaptic and postsynaptic membranes were labeled with antibodies against Horseradish peroxidase (HRP,gray) and Discs-large (Dlg, green). Ghost boutons (GBs) (arrow) are identified by the lack of Dlg. Scale bar is 10 µm. **c,** Zoom of GBs examples for control showing MyoII recruitment and clustering. **d,** Zoom of GBs examples for neuronal expression of Zip-RNAi without noticeable myosin, suggesting that the K/D was effective. **c, d,** Scale bar is 2 µm. Representative images from 9 NMJs/6 larvae (NSyb-Gal4>+) and 12 NMJs/2 larvae (NSyb-Gal4>Zip-RNAi) from 1 experiment.

Supplementary Figure 21

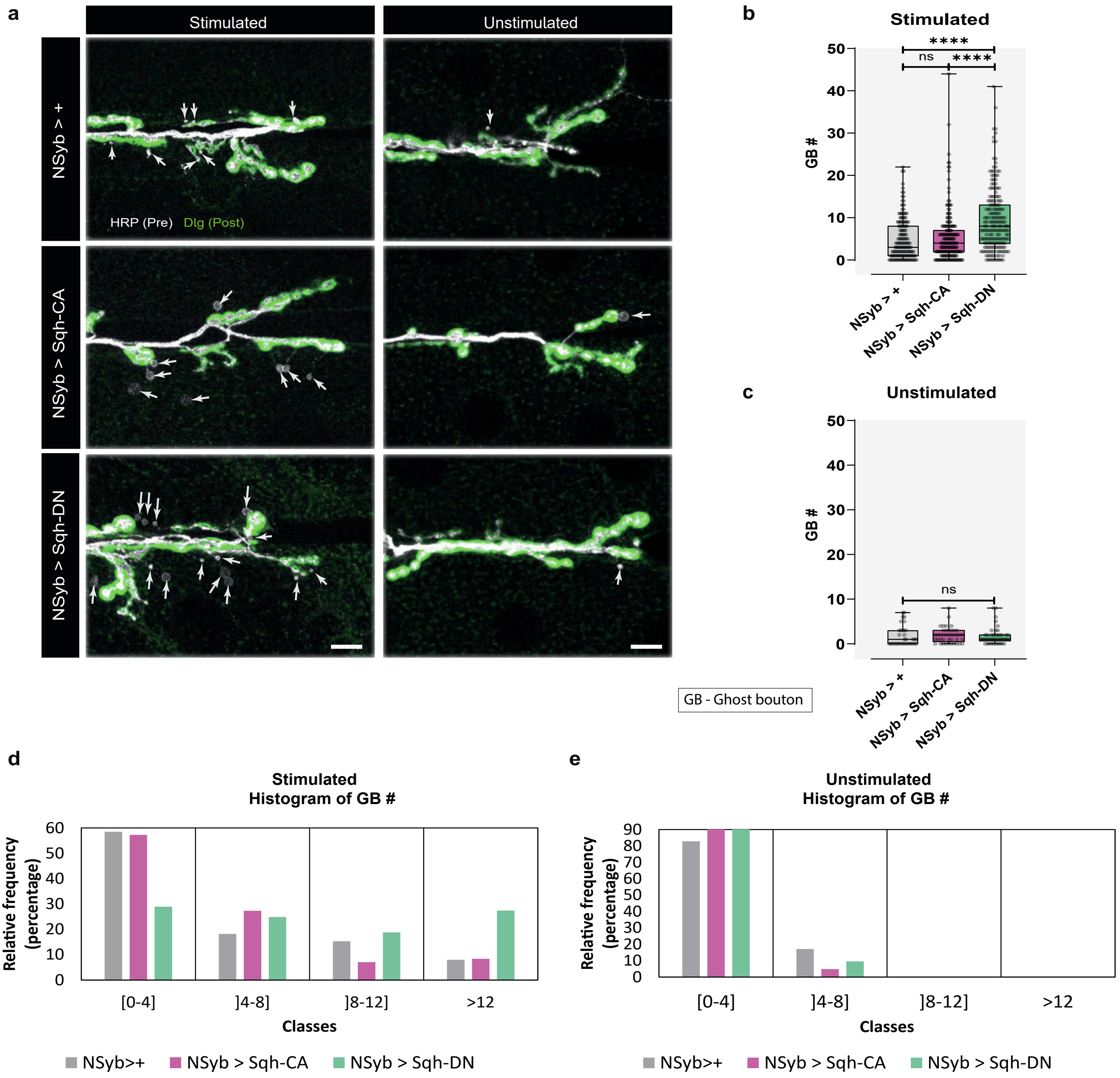

Supplementary Figure 21. Effects of altering activity of MyoII on activity-dependent plasticity. **a,** Representative image of control animals and animals expressing constitutively active (CA) or dominant negative (DN) forms of non-muscle myosin-II (MyoII) with (left) or without (right) high-K^+ stimulation. We used NSyb-Gal4 (pan-neuronal driver) to express phosphomimetic Spaghetti squash (Sqh)-CA or non-phosphorylatable Sqh-DN in MNs. The presynaptic and postsynaptic membranes were labeled with antibodies for Horseradish peroxidase (HRP, gray) and Discs-large (Dlg, green), respectively. Ghost boutons (GBs) (arrow) are identified by the lack of Dlg. Scale bar, 10 µm. **b,** Boxplot (min to max) showing GB number after stimulation for larvae expressing Sqh-CA or Sqh-DN in neurons and control larvae (NSyb>+). We found that neuronal expression of Sqh-DN, but not of Sqh-CA, increased GB number after acute stimulation compared to the control. Line is at median. All data points are presented. N=176 NMJs (NSyb-Gal4>+), 226 (NSyb-Gal4>Sqh-CA) and 196 (NSyb-Gal4>Sqh-DN), from 5 biologically independent experiments. **c,** Boxplot (min to max) showing GB number without stimulation for larvae expressing Sqh-CA or Sqh-DN in neurons and control larvae (NSyb>+). N=35 NMJs (NSyb-Gal4>+), 41 (NSyb-Gal4>Sqh-CA) and 42 (NSyb-Gal4>Sqh-DN), and 3 biologically independent experiments. **d, e,** Histograms showing the percentage of GBs in each class for stimulated (d) and unstimulated (e) NMJs. Boxplots show 25th–75th percentiles, lines at the median, and whiskers from minimum to maximum. Statistical significance was determined with non-parametric Kruskal-Wallis test (two-tailed);****p<0.0001, ns is not significant. Source data provided as a Source Data file.

Supplementary Figure 22

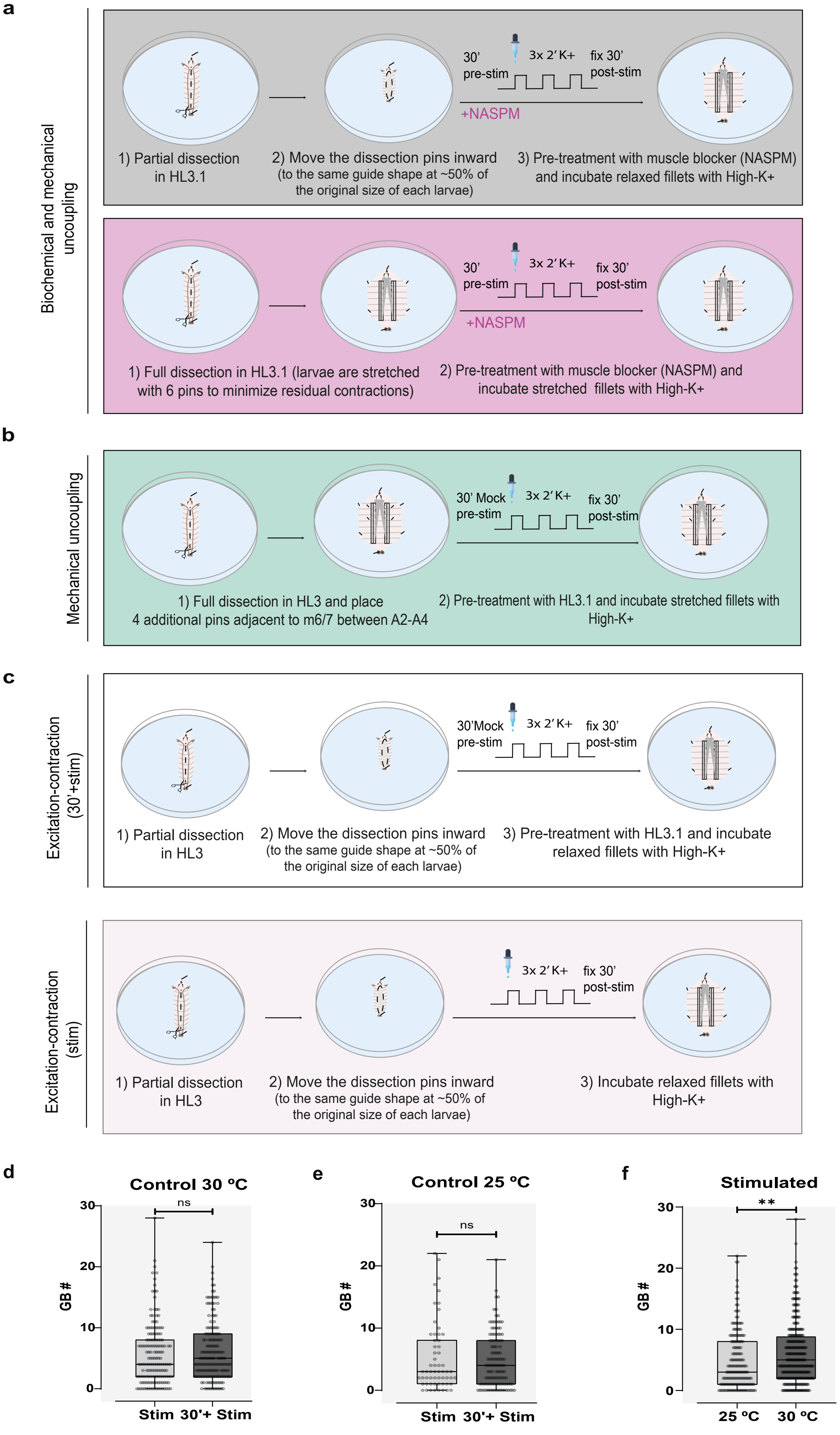

GB - Ghost bouton

Supplementary Figure 22. Experimental design for blocking muscle contractions during activity-dependent plasticity. **a-c,** Schematics for stimulation protocols designed to uncouple excitation-contraction and the stimulation controls (30+ stim) that were performed in parallel for each experiment. **a,b,** Strategies adopted to uncouple MN excitability from muscle contraction: muscle blocker (a) vs. mechanical stretching (b) larvae. **a,** 1-Naphytlacetyl Spermine Trihydrochloride (NASPM, glutamate receptor -GluR-antagonist) was used inhibit GluRs therefore promoting both biochemical and mechanical uncoupling of excitation-contraction cycles. We confirmed the effect of the drug on blocking muscle contraction using relaxed larvae (top). For further experiments, larvae were fully stretched with 6 pins (bottom) to minimize residual contraction and to verify drug efficacy (NMJs that contracted significantly exhibited visible muscle damage or tearing). **b,** To mechanically block muscle contraction and simultaneously avoid preparation tearing larvae were stretched with 10 pins (an additional 4 pins were placed surrounding the NMJ 6/7 between A2-A4). **c,** Comparison between controls pretreated with HL3 mock solution (30+stim, up) and normal controls (stim, bottom) used for stimulation. **d,e,** Boxplot (min to max) showing ghost bouton (GB) numbers for 30+stim and stim protocols in control (NSyb > +) animals raised at 30ºC (e) or 25ºC (e). **f,** Boxplot (min to max) to show GB number in control animals raised at temperatures of 25 or 30ºC. We found that GB number in larvae raised at 30ºC was increased compared to the ones that stayed at 25ºC. Line is at median. All data points are presented. All larvae were of the genotype NSyb-Gal4>+. N of NMJs: **d,** 159 (Stim 30ºC), 154 (30’ + Stim 30ºC); **e,** 59 (Stim 25ºC) and 117 (30’ + Stim 25ºC); **f,** 176 (25ºC) and 313 (30ºC), from 5 biologically independent experiments. Boxplots show 25th–75th percentiles, lines at the median, and whiskers from minimum to maximum. Statistical significance was determined with non-parametric Kruskal-Wallis test (two-tailed);****p<0.0001, ns is not significant. Source data provided as a Source Data file.

Supplementary Figure 23

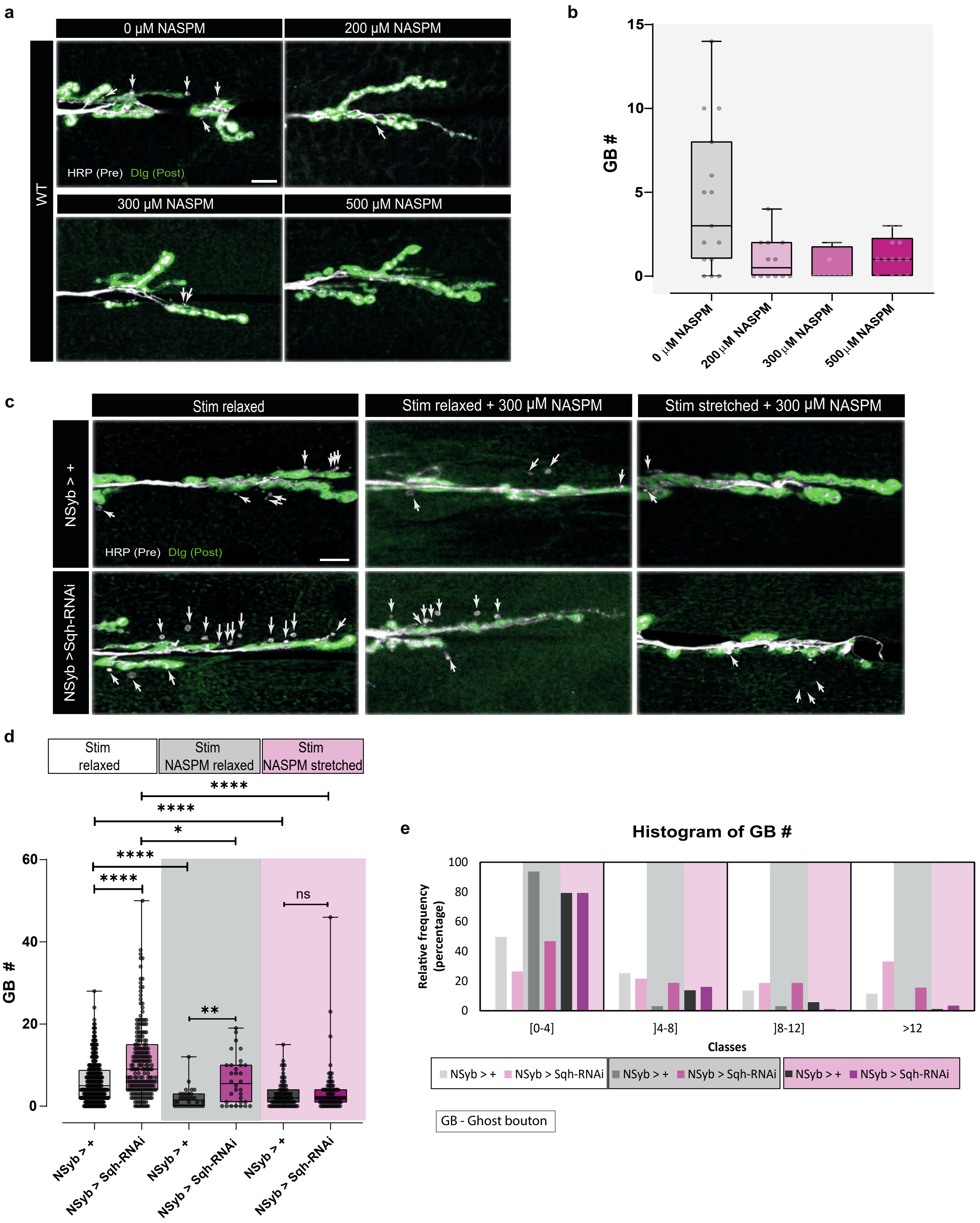

Supplementary Figure 23. Effects of blocking muscle activation on activity-dependent bouton formation with MyoII knockdown. a,b, Effects of blocking muscle activation with 1-Naphtylacetil spermine trihydrochloride (NASPM, glutamate receptor antagonist) on activity-dependent bouton formation. **a,** Representative NMJ images of WT larvae (W^{1118}) incubated with different concentrations of NASPM (indicated in the figure) and stimulated with high K^+. **b,** Boxplot (min to max) showing GB numbers after stimulation (Stim relaxed) with 0, 200, 300, or 500 µM NASPM. N=15 (0µM NASPM), 12 (200µM NASPM), 12 (300µM NASPM) and 14 (500µM NASPM). **c,** Representative NMJs images of control and MyoII K/D larvae treated with 0 or 300 µM NASPM and stimulated with high-K^+. The presynaptic and postsynaptic membranes were labeled with antibodies for Horseradish peroxidase (HRP, gray) and Discs-large (Dlg, green), respectively. Ghost boutons (GBs) (arrow) are identified by the lack of Dlg. Scale bar, 10 µm. **d,** Boxplot (min to max) showing GB numbers after stimulation with 300 µM NASPM. We found that blocking muscle depolarization, and subsequent contraction, with 300 µM NASPM reduced bouton formation in neuronal Sqh-RNAi expressing and control larvae. Line is at median. All data points are presented. **e,** Histogram showing the percentage of GBs in HL3.1 or 300 µM NASPM. Stim relaxed N= 322 (NSyb-Gal4>+), 208 (NSyb-Gal4>Sqh-RNAi); Stim relaxed + 300µM NASPM N= 32 (NSyb-Gal4>+), 32 (NSyb-Gal4 > Sqh-RNAi); Stim stretched + 300µM NASPM N= 88 (NSyb-Gal4>+), 87 (NSyb-Gal4> Sqh-RNAi) from 5 biologically independent experiments. Boxplots show 25th–75th percentiles, lines at the median, and whiskers from minimum to maximum. Statistical significance was determined with non-parametric Kruskal-Wallis test (two-tailed);****$p<0.0001$, *$p<0.05$, ns is non-significant. P-value NSyb>+ (NASPM Relaxed) vs. NSyb > Sqh-RNAi (NASPM Relaxed)=0.006; NSyb > Sqh-RNAi (Stim Relaxed) vs NSyb > Sqh-RNAi (NASPM Relaxed)=0.0245. Source data provided as a Source Data file.

Supplementary Figure 24

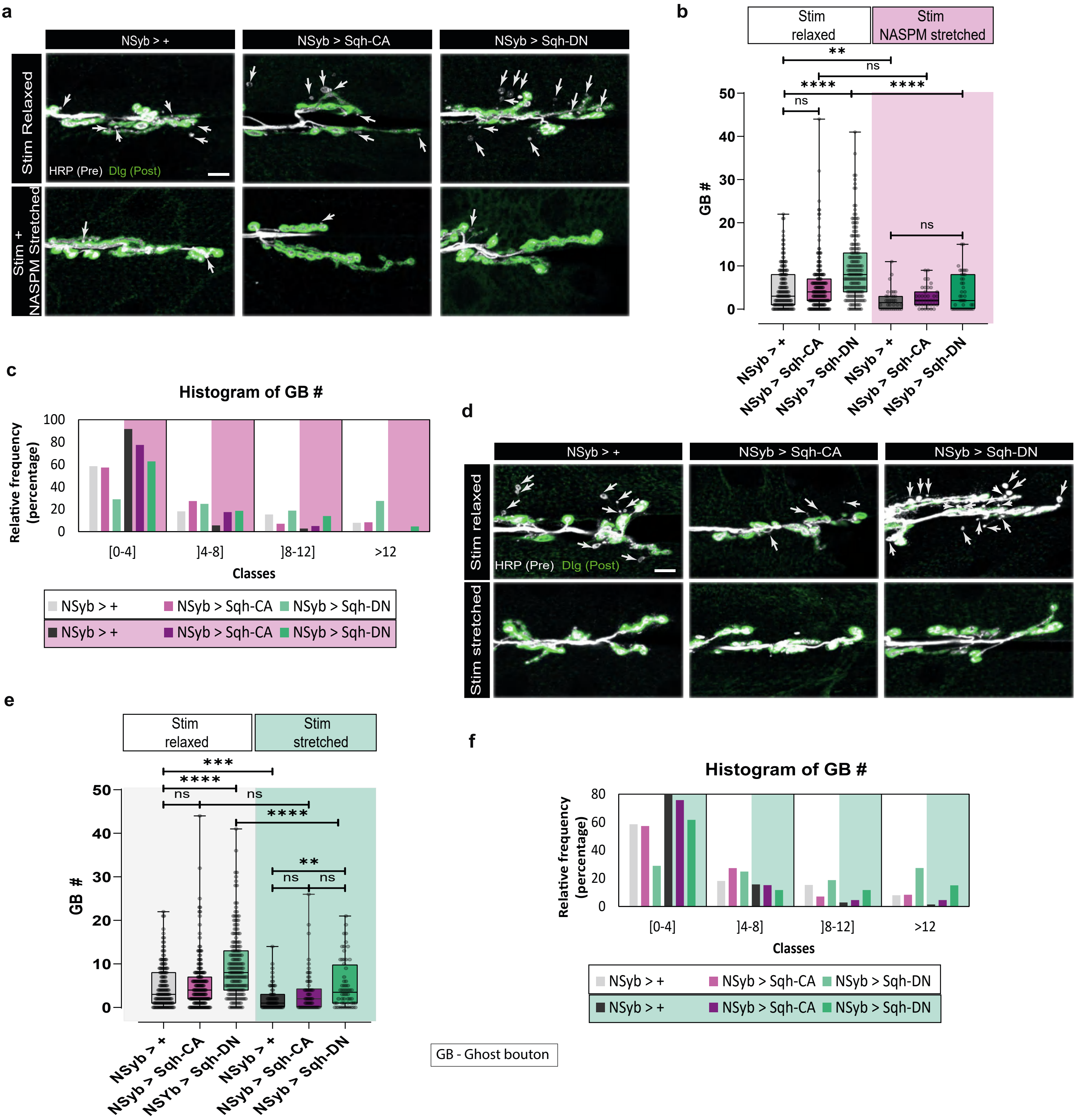

Supplementary Figure 24. Effects of blocking muscle activation and/or contraction on activity-dependent bouton formation with MyoII activation or inactivation. **a,** Representative NMJ images of larvae expressing constitutively active or inactive non-muscle myosin-II (MyoII) – Spaghetti squash (Sqh)-CA or Sqh-DN respectively – in neurons and control larvae (NSyb > +) that were incubated with 300 µM 1-Naphtylacetil spermine trihydrochloride (NASPM, glutamate receptor antagonist) and stimulated with high-K^+. **b,** Boxplot (min to max) of GB number. Blocking muscle depolarization, and subsequent contraction, with 300µM NASPM decreased stimulation induced bouton formation both in control and neuronal expressing Sqh-DN larvae, but not in neuronal expressing Sqh-CA larvae. Line is at median. All data points are presented. **P-value Nsyb relaxed vs NASPM=0.0065 **c,** Histogram of percentage of ghost bouton (GB) numbers in each class for animals treated with HL3.1 or NASPM. Stim relaxed N= 176 (NSyb-Gal4>+), 226 (NSyb-Gal4 > Sqh-CA), 196 (NSyb-Gal4 > Sqh-DN); Stim stretched + 300µM NASPM N= 36 (NSyb-Gal4>+), 40 (NSyb-Gal4 > Sqh-CA), 43 (NSyb-Gal4>Sqh-DN), from 5 biologically independent experiments. **d,** Representative NMJ images of larvae expressing Sqh-CA or Sqh-DN in neurons and control larvae (NSyb > +) that were stimulated with high-K^+ relaxed or mechanically stretched. **e,** Box-plot (min to max) of GB number. Blocking muscle contraction by mechanically stretching the larvae also decreased bouton formation upon stimulation in control and neuronal expressing Sqh-DN larvae, but not in neuronal expressing Sqh-CA larvae. Line is at median. All data points are presented. *P-value NSyb>+ relaxed vs stretched=0.0002, **p-value NSyb>+ vs nSyb>SqhDN=0.0098. **f,** Histogram of percentage of GBs in each class for the animals that were stimulated relaxed or stretched. Stim relaxed N= 176 (NSyb-Gal4>+), 226 (NSyb-Gal4>Sqh-CA), 196 (NSyb-Gal4 > Sqh-DN); Stim stretched N= 70 (NSyb-Gal4>+), 66 (NSyb-Gal4 > Sqh-CA), 60 (NSyb-Gal4 > Sqh-DN), from 5 biologically independent experiments. The presynaptic and postsynaptic membranes were labeled with antibodies for Horseradish peroxidase (HRP, gray) and Discs-large (Dlg, green), respectively. GBs (arrow) are identified by the lack of Dlg. Scale bar in NMJs are 10 µm. Boxplots show 25th–75th percentiles, lines at the median, and whiskers from minimum to maximum. Statistical significance was determined with non-parametric Kruskal-Wallis test (two-tailed);****p<0.0001,***p<0.001, **p<0.01, ns is not significant. Source data provided as a Source Data file.

Supplementary Figure 25

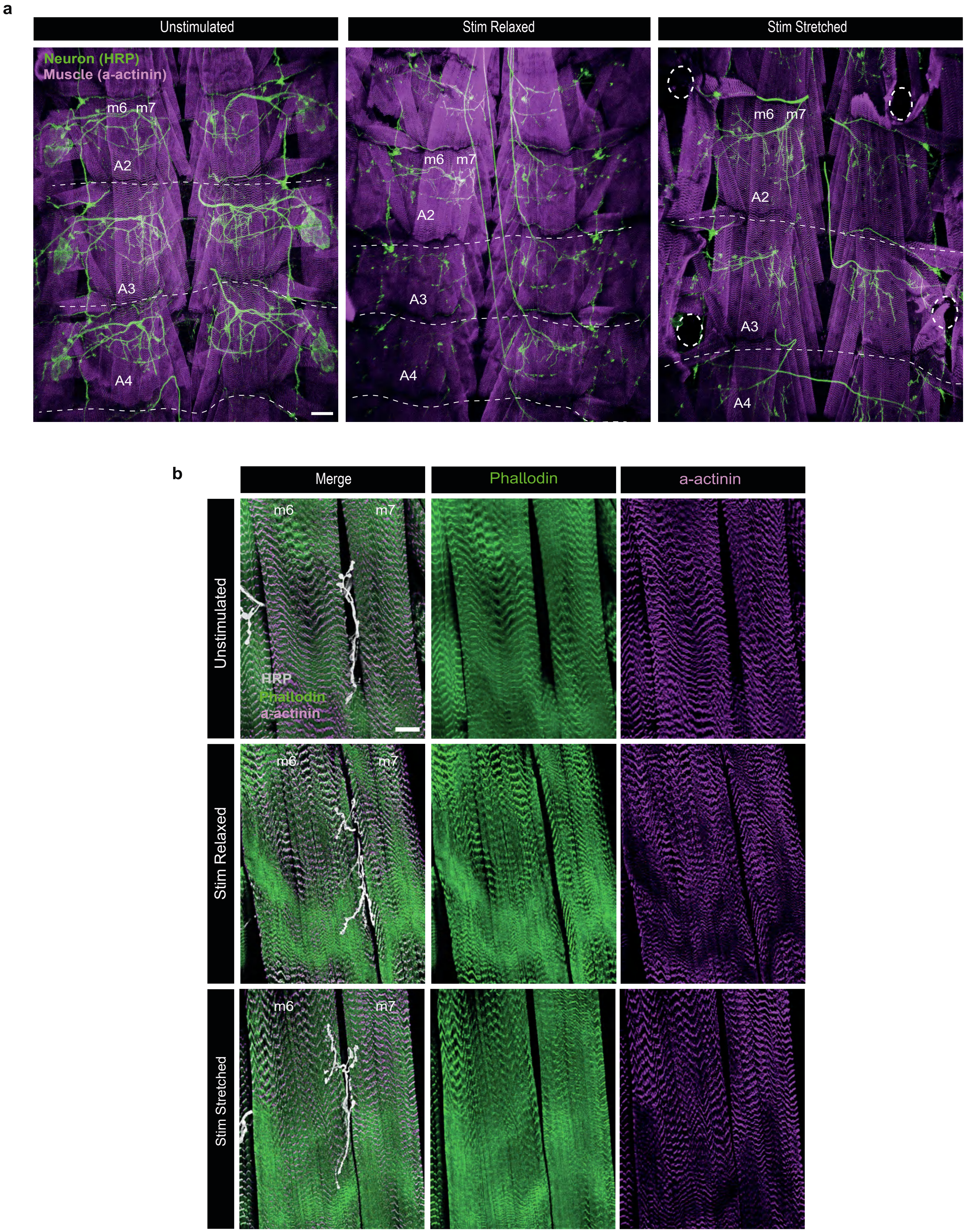

Supplementary Figure 25. *Drosophila* larval body wall muscles maintain their integrity after larval stimulation in the conditions relaxed and stretched. a, Representative images showing the body-wall muscles (magenta) for unstimulated, stimulated/relaxed and stimulated/stretched larvae. Muscles were visualized by antibody staining against α-actinin, and imaging shows the ventral muscle groups from segments A2-A4, with muscles 6/7 indicated by m6 and m7 in the image. The neuronal membranes were labeled with antibody for Horseradish peroxidase (HRP, green). Circular ROIs show openings left by insect pins used for mechanical stretching. Scale bar is 50 µm. **b,** Representative images of ventral longitudinal muscles 6,7 of the 3rd abdominal segment (A3) for each condition represented, of WT larvae (W^{1118}) exposed to different degrees of stretching. Muscle structural integrity was examined using phalloidin (green) staining of actin in the body wall muscles for each condition. α-actinin from Z-lines was used to assess the structural integrity of sarcomeres. Neuronal membrane was labeled with antibody for Horseradish peroxidase (HRP, gray). Scale bar is 10 µm. Data are representative of at least 3 biologically independent experiments (n=3 larvae and 3 NMJs for unstimulated larvae and n=6 larvae and 12 NMJs for stimulated larvae, both for relaxed and stretched).
