# Supplementary material for: Drosophila motor neuron boutons remodel through membrane blebbing coupled with muscle contraction
Source: Nat Commun. 2023 Jun 8;14:3352. doi: 10.1038/s41467-023-38421-9 (PMC10250368; doi:10.1038/s41467-023-38421-9)
Supplement: Supplementary file 3 — Description to Additional Supplementary Files [file 41467_2023_38421_MOESM3_ESM.pdf]

Description of Additional Supplementary Files

Supplementary Tables 1– 3 (excel files within data source file)

Supplementary Table 1 – List of stocks used in this study

Supplementary Table 2 – Recombinants and other compound lines used

Supplementary Table 3 – List of antibodies, dilution and fixatives used

Supplementary Movies 1 – 19:

Supplementary Movie 1. Bouton formation in WT after high-K^+: fast bouton formation (together with muscle contraction). Example of a time-lapse movie of fast bouton formation after high-K^+ stimulation, associated with visible muscle contraction. Neuronal membranes were labeled with UAS-CD4-Tom under the control of NSyb-Gal4 (pan-neuronal driver). Arrow indicates where the bouton emerges.

Supplementary Movie 2. Bouton formation in WT after high-K^+: slow bouton formation. Example of a time-lapse movie of slow bouton formation after high-K^+ stimulation with no visible muscle contraction. Neuronal membranes were labeled with UAS-CD4-Tom under the control of NSyb-Gal4 (pan-neuronal driver). Arrow indicates where the bouton emerges.

Supplementary Movie 3. Bouton formation with optogenetic stimulation: fast bouton formation. Time-lapse movie of bouton formation after 3xCsChR stimulation using red light. Neuronal membranes were labeled with UAS-CD4-Tom under the control of OK6-Gal4 (a MN driver). Arrow indicates where the bouton emerges.

Supplementary Movie 4. Bouton formation with optogenetics: slow bouton formation. Time-lapse movie of bouton formation after 3xCsChR stimulation using red light. Neuronal membranes were labeled with UAS-CD4-Tom under the control of OK6-Gal4 (a MN driver). Arrow indicates where the bouton emerges.

Supplementary Movie 5. Bouton formation without stimulation. Example of a time-lapse movie of bouton formation without stimulation (at rest). Neuronal membranes were labeled with UAS-CD4-Tom under the control of NSyb-Gal4 (pan-neuronal driver). Arrow indicates where the bouton emerges.

Supplementary Movie 6. Bouton showing the bleb life phases after high-K^+ stimulation. Time-lapse movie of GB formation induced by high-K^+ stimulation showing all bleb life phases, including stabilization. Neuronal membrane and F-actin were labeled with UAS-CD8-GFP and UAS-Lifeact-Ruby, both under the control of NSyb-Gal4. Arrow indicates where the bouton emerges.

Supplementary Movie 7. Sequential bouton formation (without retraction) after high-K^+ stimulation. Time-lapse movie of GB formation induced by high-K^+ stimulation showing an example of sequential bouton formation without retraction. Neuronal membrane and F-actin were labeled with UAS-CD4-GFP and UAS-Lifeact-Ruby, both under the control of NSyb-Gal4. Arrow indicates where the bouton emerges.

Supplementary Movie 8. Sequential bouton formation (with retraction) after high-K^+ stimulation. Time-lapse movie of GB formation induced by high-K^+ stimulation showing an example of sequential bouton formation with retraction. Neuronal membrane and F-actin were labeled with UAS-CD4-GFP and UAS-Lifeact-Ruby, both under the control of NSyb-Gal4. Arrow indicates where the bouton emerges.

Supplementary Movie 9. Bouton showing bleb phases without stimulation (at rest). Time-lapse movie of GB formation without stimulation (at rest) showing an example of bouton formation with all bleb phases. Neuronal membrane and F-actin were labeled with UAS-CD4-GFP and UAS-Lifeact-Ruby, both under the control of NSyb-Gal4. Arrow indicates where the bouton emerges.

Supplementary Movie 10. Example #2 of bouton blebbing unstimulated. Time-lapse movie of GB formation without stimulation (at rest). Neuronal membrane and F-actin were labeled with UAS-CD4-GFP and UAS-Lifeact-Ruby, both under the control of NSyb-Gal4. Arrow indicates where the bouton emerges.

Supplementary Movie 11. Example of bouton remodeling unstimulated. Time-lapse movie of GB remodeling without stimulation (at rest) showing acting flux concomitant with bouton remodeling. Neuronal membrane and F-actin were labeled with UAS-CD4-GFP and UAS-Lifeact-Ruby, both under the control of NSyb-Gal4. Arrow indicates where the bouton emerges.

Supplementary Movie 12. Example #2 of bouton remodeling unstimulated. Time-lapse movie of GB remodeling without stimulation (at rest) showing acting flux concomitant with bouton remodeling. Neuronal membrane and F-actin were labeled with UAS-CD4-GFP and UAS-Lifeact-Ruby, both under the control of NSyb-Gal4. Arrow indicates where the bouton emerges.

Supplementary Movie 13. FRAP analysis of F-actin reporters Lifeact and Actin5C. Representative movie of a bouton co-expressing Actin5C-GFP (green) and Lifeact-Ruby (magenta) (under control of NSyb-Gal4), which were photobleached and allowed to recover for 300s. The white circle indicates the ROI for photobleaching.

Supplementary Movie 14. F-actin recovery dynamics after FRAP: HL3.1 VS DMSO VS JAS. Time-lapse movies showing Lifeact-GFP expression in the presynaptic terminals during FRAP. Photobleaching of F-actin probe in synaptic boutons to examine actin dynamics after JAS treatment, compared to controls (HL3.1 and DMSO). White circles indicate photobleaching ROI.

Supplementary Movie 15. MyoII punctum preceding bouton formation. Time-lapse movie of MyoII dynamics during bouton formation, showing an example of a MyoII punctum at the base preceding the bouton formation. Neuronal morphology and MyoII were visualized with LexAop-mCherry under the control of the LexA driver DvGlut, and to label MyoII we used GFP-tagged Sqh (light chain) in a Sqh-null background (SqhAX3). Arrow indicates where the bouton emerges.

Supplementary Movie 16. MyoII flow during bouton expansion. Time-lapse movie of MyoII dynamics during bouton formation, showing an example of a bouton forming with MyoII flow to the new bouton during the expansion phase. Neuronal morphology and MyoII were visualized with LexAop-mCherry under the control of the LexA driver DvGlut, and to label MyoII we used GFP-tagged Sqh (light chain) in a Sqh-null background (SqhAX3). Arrow indicates where the bouton emerges.

Supplementary Movie 17. MyoII accumulation during bouton remodeling. Time-lapse movie of bouton remodeling showing MyoII dynamics and accumulations leading to clear changes in bouton morphology. Neuronal morphology and MyoII were visualized with LexAop-mCherry under the control of the LexA driver DvGlut, and to label MyoII we used GFP-tagged Sqh (light chain) in a Sqh-null background (SqhAX3). Arrow indicates where the accumulations occur.

Supplementary Movie 18. Bouton formation in neuronal MyoII K/D. Time-lapse movie of bouton formation when MyoII was K/D in neurons. We observed formation of bouton threads associated with visible muscle contractions. NSyb-Gal4 was used to drive UAS-CD4-Tom (membrane label) and UAS-Sqh-RNAi expression. Arrow indicates where the bouton emerges.

Supplementary Movie 19. Live analysis of muscle contraction blockade with larval stretching. Time-lapse movie of larvae subjected to different degrees of stretching and stimulation. Same larvae is imaged in the different conditions. Left to right: 1) HL3.1 relaxed (unstimulated), 2) high-K^+ relaxed (stimulated relaxed) and 3) high-K^+ stretched (stimulated stretched). Muscles were visualized by expression of Tropomyosin-GFP (protein trap), focusing on ventral muscles in segments A2-A4.
