# Supplementary material for: Drosophila motor neuron boutons remodel through membrane blebbing coupled with muscle contraction
Source: Nat Commun. 2023 Jun 8;14:3352. doi: 10.1038/s41467-023-38421-9 (PMC10250368; doi:10.1038/s41467-023-38421-9)
Supplement: Supplementary file 23 — Reporting Summary [file 41467_2023_38421_MOESM23_ESM.pdf]

nature portfolio

Corresponding author(s): Rita O. Teodoro

Last updated by author(s): Apr 12, 2023

Reporting Summary

Nature Portfolio wishes to improve the reproducibility of the work that we publish. This form provides structure for consistency and transparency in reporting. For further information on Nature Portfolio policies, see our Editorial Policies and the Editorial Policy Checklist.

Statistics

For all statistical analyses, confirm that the following items are present in the figure legend, table legend, main text, or Methods section.

n/a	Confirmed	
☐	☒	The exact sample size (*n*) for each experimental group/condition, given as a discrete number and unit of measurement
☐	☒	A statement on whether measurements were taken from distinct samples or whether the same sample was measured repeatedly
☐	☒	The statistical test(s) used AND whether they are one- or two-sided<br>*Only common tests should be described solely by name; describe more complex techniques in the Methods section.*
☒	☐	A description of all covariates tested
☐	☒	A description of any assumptions or corrections, such as tests of normality and adjustment for multiple comparisons
☐	☒	A full description of the statistical parameters including central tendency (e.g. means) or other basic estimates (e.g. regression coefficient) AND variation (e.g. standard deviation) or associated estimates of uncertainty (e.g. confidence intervals)
☐	☒	For null hypothesis testing, the test statistic (e.g. *F*, *t*, *r*) with confidence intervals, effect sizes, degrees of freedom and *P* value noted<br>*Give P values as exact values whenever suitable.*
☒	☐	For Bayesian analysis, information on the choice of priors and Markov chain Monte Carlo settings
☒	☐	For hierarchical and complex designs, identification of the appropriate level for tests and full reporting of outcomes
☒	☐	Estimates of effect sizes (e.g. Cohen's *d*, Pearson's *r*), indicating how they were calculated

Our web collection on statistics for biologists contains articles on many of the points above.

Software and code

Policy information about availability of computer code

Data collection	Microscopy: Zeiss LSM 710 or LSM 980 (super resolution images with Airyscanning) laser scanning confocal and Andor Spinning Disk<br>Software: Black 2011 SP1 for LSM 710, ZEISS ZEN 3.3 (blue edition) for LSM 980 and Andor iQ3 (3.6) for Spinning disk
Data analysis	Microscopy: Image J v2.p52, Fiji 3, Images assembled in Adobe Illustrator (2022 version 26.0.1) and Adobe Photoshop (2020 version 21.2.1)<br>Graphs and Statistics: Microsoft Excel v. 16.70, GraphPad Prism version 8.0.1 and version 8.0.2

For manuscripts utilizing custom algorithms or software that are central to the research but not yet described in published literature, software must be made available to editors and reviewers. We strongly encourage code deposition in a community repository (e.g. GitHub). See the Nature Portfolio guidelines for submitting code & software for further information.

Data

Policy information about availability of data

All manuscripts must include a data availability statement. This statement should provide the following information, where applicable:

- Accession codes, unique identifiers, or web links for publicly available datasets
- A description of any restrictions on data availability
- For clinical datasets or third party data, please ensure that the statement adheres to our policy

The authors declare that the data supporting the findings of this study are available within the paper and supplementary information files.

Human research participants

Policy information about studies involving human research participants and Sex and Gender in Research.

Reporting on sex and gender	N/A
Population characteristics	N/A
Recruitment	N/A
Ethics oversight	N/A

Note that full information on the approval of the study protocol must also be provided in the manuscript.

Field-specific reporting

Please select the one below that is the best fit for your research. If you are not sure, read the appropriate sections before making your selection.

☒ Life sciences ☐ Behavioural & social sciences ☐ Ecological, evolutionary & environmental sciences

For a reference copy of the document with all sections, see nature.com/documents/nr-reporting-summary-flat.pdf

Life sciences study design

All studies must disclose on these points even when the disclosure is negative.

Sample size	Sample sizes were chosen as established in the field; sizes are typically based on the following: use 6 larvae per genotype per experiment; from these, we collect up to 6 NMJ per larvae for fixed imaging, and 1 NMJ per larvae for live imaging. If bouton diameter is to be quantified, we quantify all boutons per NMJ. We never exclude animals or NMJs (except if integrity is disrupted). We also confirmed that the number of animals chosen was similar or higher than what other similar studies use, publications such as: https://doi.org/10.1038/s41467-023-36644-4, https://doi.org/10.1038/s41467-022-31253-z, https://doi.org/10.1038/s41467-022-35417-9
Data exclusions	No data was excluded
Replication	The number of animals and data points for each experiment are stated in each figure legend. Each figure shows all data points collected and analyzed. Unstimulated samples showed low variation and were always reproducible. For stimulation experiments higher variation was observed. In these cases, biologically independent experiments were either statistically different per experiment or showed a nonstatistical trend towards the significant changes observed in the distribution of data seen across all experiments. All attempts at replication were successful in the immunostaining experiments.<br><br>In all experiments at least 3 replicates (6 animals per experiment/condition) were done except for antibody and protein trap stainning in which we performed at least 2 replicates. The exact number of specimens or NMJs analyzed are provided in the Methods Section and in supplementary material as a Table (Table 4).
Randomization	This is not relevant to our study because we compared samples with different genetic backgrounds. Still, whenever a comparison was made between a treatment or genotype and a control group, experiments were always ran in parallel and processed in the same tube. Order of genotypes/treatment, acquisition and analysis of data was performed in an interleaved manner.
Blinding	There was no blinding during group allocation because our study samples were not allocated to groups.

Reporting for specific materials, systems and methods

We require information from authors about some types of materials, experimental systems and methods used in many studies. Here, indicate whether each material, system or method listed is relevant to your study. If you are not sure if a list item applies to your research, read the appropriate section before selecting a response.

Materials & experimental systems

n/a	Involved in the study
☐	☒ Antibodies
☒	☐ Eukaryotic cell lines
☒	☐ Palaeontology and archaeology
☐	☒ Animals and other organisms
☒	☐ Clinical data
☒	☐ Dual use research of concern

Methods

n/a	Involved in the study
☒	☐ ChIP-seq
☒	☐ Flow cytometry
☒	☐ MRI-based neuroimaging

Antibodies

Antibodies used

Primary antibodies: mouse anti-Dlg (4F3;1:250; Developmental Studies Hybridoma Bank; University of Iowa, Iowa, IA, USA, AB_528203), α-actinin (2G3-3D7; Developmental Studies Hybridoma Bank), AB_2721943 , rabbit anti-GFP (1:1000; Thermofischer A11122). The following antibodies were used and obtained as gifts since they are not commercially available: rabbit anti-Dlg (1:10000; Vivian Budnik lab), rabbit anti-Myosin-II, zipper (1:1000, Christine Field); rat anti-Filamin (1:800; Mirka Uhlivora lab). All antibodies were previously validated (see the next section).

All secondary antibodies used in this study were purchased from Jackson Immunoresearch and used at 1:500 (initial dilution in 50% glycerol, according to manufacturer indications): A488/RhRx/A647 donkey anti-mouse (used for mouse anti-Dlg and mouse anti-α-actinin), A488/A647 donkey anti rabbit (used for GFP or myosin-II), A488 donkey anti-Rabbit XR (used for anti-mouse Dlg in filamin stainning) and Cy3 donkey anti-Rat (used for filamin). All with minimum cross-reactivity for all relevant species. Also from Jackson Immunoresearch Horseradish peroxidase (HRP) conjugated to Cy3, Alexa488, Alexa647 or Alexa405 was used to label neuronal membrane (1:500).

Validation

These antibodies were described and validated in previous published papers:
Validation for commercially available antibodies is described on manufacturer's websites, and confirmed in multiple studies:
Mouse anti-Dlg: https://dshb.biology.uiowa.edu/4F3-anti-discs-large
Rabbit anti-GFP: https://www.thermofisher.com/antibody/product/GFP-Antibody-Polyclonal/A-11122
Secondary and HRP-conjugated antibodies: www.jacksonimmuno.com
Filamin antibody: Kulshammer and Uhlirova. J. Cell Sci. 2013, and subsequently used in other publications
Rabbit anti-Dlg: Budnik et al. Neuron 1996, and subsequently used in hundreds of publications.
Rabbit anti-Myosin II: Fields and Alberts, Journal of Cell Biology, 1995; subsequently used in several other publications.
Mouse anti-α-actinin: Pardue ML, Journal of Cell Biology, 1989; subsequently used in several other publications.

Animals and other research organisms

Policy information about studies involving animals; ARRIVE guidelines recommended for reporting animal research, and Sex and Gender in Research

Laboratory animals

Drosophila melanogaster 3rd instar larvae were used in this study. In Supplementary Fig. 5 2ns instar larvae were also used. Strains (and their origin) used is this study are:
Stocks obtained from the Bloomington Drosophila Stock Center (BDSC): UAS-Zip-RNAi (#36727, #37480) UAS-Sqh RNAi (#32439, #31542, #7916, #38222), UAS-Sqh-CA (#64411), UAS-Sqh-DN (#64114), UAS-CD4-Tom (#35837), UAS-CD4-GFP (#35836), NSyb-Gal4 (from Thomas L. Schwarz Lab, #51635), OK6-Gal4 (from O'Kane lab, #64199), UAS-Act5C-GFP (#7309), UAS-BRP-GFP(#36292), Trpomyosin-GFP trap (#51537), sqhAX3;;SqhGFP (#57144), 13XLexAop-6XmCherry-HA (#52271), UAS-Lifeact-Ruby (Ivo Telley lab gift), UAS-Lifeact-GFP (Ivo Telley lab gift). From the Vienna Drosophila Resource Center (VDRC): GluRIIE (flyfos) VDRC#318061, Dlg (flyfos) VDRC#318133, Sqh (flyfos) VDRC#318487. From the Kyotto Resource Center: Zip-GFP (trap) Kyotto#115082. DV-Glut-LexA described in this study, see methods for details).

Wild animals

No wild animals were used in this study.

Reporting on sex

Male and female third instar larvae were used interchangeably of the genotypes described in the methods, text and figures. When sqhAX3;;SqhGFP larvae were used, males were selected based on X-chromosome presence of the mutation (only males were mutant).

Field-collected samples

No field collected samples were used in this study.

Ethics oversight

This study did not require an ethical approval since laboratory animals were insects.

Note that full information on the approval of the study protocol must also be provided in the manuscript.
